# The negative consequences of sports betting opportunities on human capital formation: Evidence from Spain

**DOI:** 10.1371/journal.pone.0258857

**Published:** 2021-10-27

**Authors:** Mar Espadafor, Sergi Martínez

**Affiliations:** Department of Social and Political Sciences, European University Institute, Fiesole, Italy; University of South Florida, UNITED STATES

## Abstract

The proliferation of on-site betting shops has received enormous public attention, becoming one of the most alarming health policy issues in contemporary cities. However, there is little evidence on whether its growing presence nearby vulnerable populations produce social harm beyond its known adverse individual effects. This study provides new evidence on the negative societal effects of betting houses. Our research design takes advantage of a new wave of openings in Madrid (Spain), which created a sudden increase in the supply of on-site gambling. Using a differences-in-differences design, we find that new betting houses decline nearby high schools’ educational performance, especially in public schools in less advantaged areas. This effect is neither trivial nor diminishing with time. This evidence suggests that betting houses increase inequality of educational opportunities. The ubiquity of betting houses around vulnerable populations in multiple regions drives us to think that these findings have relevant policy implications for many countries currently designing policies tackling the increase of problem gambling.

## Introduction

Gambling is increasingly recognized as a social disease [1]. It is becoming one of the most relevant present-day addictions: the World Health Organization states that 350 million gamblers display problematic patterns each year [2, 3]. In their recent cross-national analysis, Calado and coauthors estimate that the problem gambling affects up to 12.3% of the OECD Countries’ population [4]. For instance, 5% of the US population has a gambling problem, despite the banning of gambling in half of the states [5]. Problem gambling is considered an addiction that, in most cases, starts during the early stages of the socialization process. One evidence of this is that, for example, the UK Gambling Commission reported that 41.4% of British young adults (15–24 years old) gambled during 2016 [6].

On the potential drivers of gambling, new evidence suggests that the contextual sphere (institutions and organizations) largely explains more variation than individual characteristics [7]. Following the Conceptual Framework of Harmful Gambling, [1, 8], the availability of different gambling opportunities is a critical factor for activating people to gamble despite its known harmfulness [9]. Accordingly, the exposure hypothesis [10, 11] points out to the current expansion of betting houses and betting shops across cities as one of the primary triggers of the growing gambling problem [12–16]. This paper further approaches the consequences of gambling opportunities’ expansion to its possible societal consequences. We focus on its effects on human capital formation and educational performance. **How does a change in the supply of betting houses and gambling opportunities affect young adults’ human capital formation process?** Despite the importance of this question, there is little empirical evidence of gambling’s impact on societal-level outcomes. This paper enters the fray by documenting the harmful effects of betting houses on high schools’ educational performance.

Extant literature on gambling splits between the causes or individual-level consequences of problem gambling. On the one hand, a growing literature analyzes the individual characteristics that draw people to addictive behaviors [7, 17–20]. It turns out that, although gambling is illegal under age 18, evidence suggests that age is an important predictor for the development of problem gambling behavior. Teenagers have access to gambling venues, and they gamble even higher proportions than adults [20, 21]. A new high school survey on drug use among adolescents confirms previous findings for the Spanish case [2]. This unexpected accessibility to gambling for minors might be explained by the fact that it is relatively easy to impersonate identity using an adult ID for online gambling and that only very few on-site betting houses in a limited number of countries effectively ask for ID cards on entry or reward cash out [2, 4, 22]. In addition, adolescents tend to have more addictive behaviors due to a weaker understanding of odds and probabilities [23], and a lack of perception of gambling as a risky activity [24]. These properties highlight adolescents’ propensity and vulnerability to an increase in the supply of gambling opportunities.

On the other hand, scholarship has paid increasing attention to the consequences of gambling in adolescence since the early 2000s due to its critical role during the socialization process (e.g., [12, 25, 26]). Most of this research strand examines the individual-level impact of gambling on adolescents and young adults, focusing on gamblers’ social integration [20], and the mental disorders derived from gambling such as other undesirable addictions like drug consumption [27–29]. Authors in this field have recently looked beyond the availability of gambling facilities, to its accessibility by minors as a contributing factor of problem gambling among adolescents [30, 31]. These studies largely focus on problem gamblers and contribute to our understanding of gambling’s underlying factors, along with the consequences of gambling on this selected population. By strictly focusing on problem gamblers, however, the societal implications remain, at large, unclear.

We believe two principal biases prevent us from extrapolating these studies’ conclusions to a broader population: the self-selection of gamblers and the accessibility to gambling facilities. First, self-selection of problem gambler’s biases, due to this group’s predisposition to gambling, any comparison or extrapolation of their behaviors to non-gamblers on any outcome. Second, and in the same vein, gambling facilities are often located in areas with more potential gamblers. Thus, previous research cannot conclude whether differences between problem gamblers and non-gamblers are due to accessibility or selection as the two groups differ in many critical confounding characteristics.

**Fig 1:**
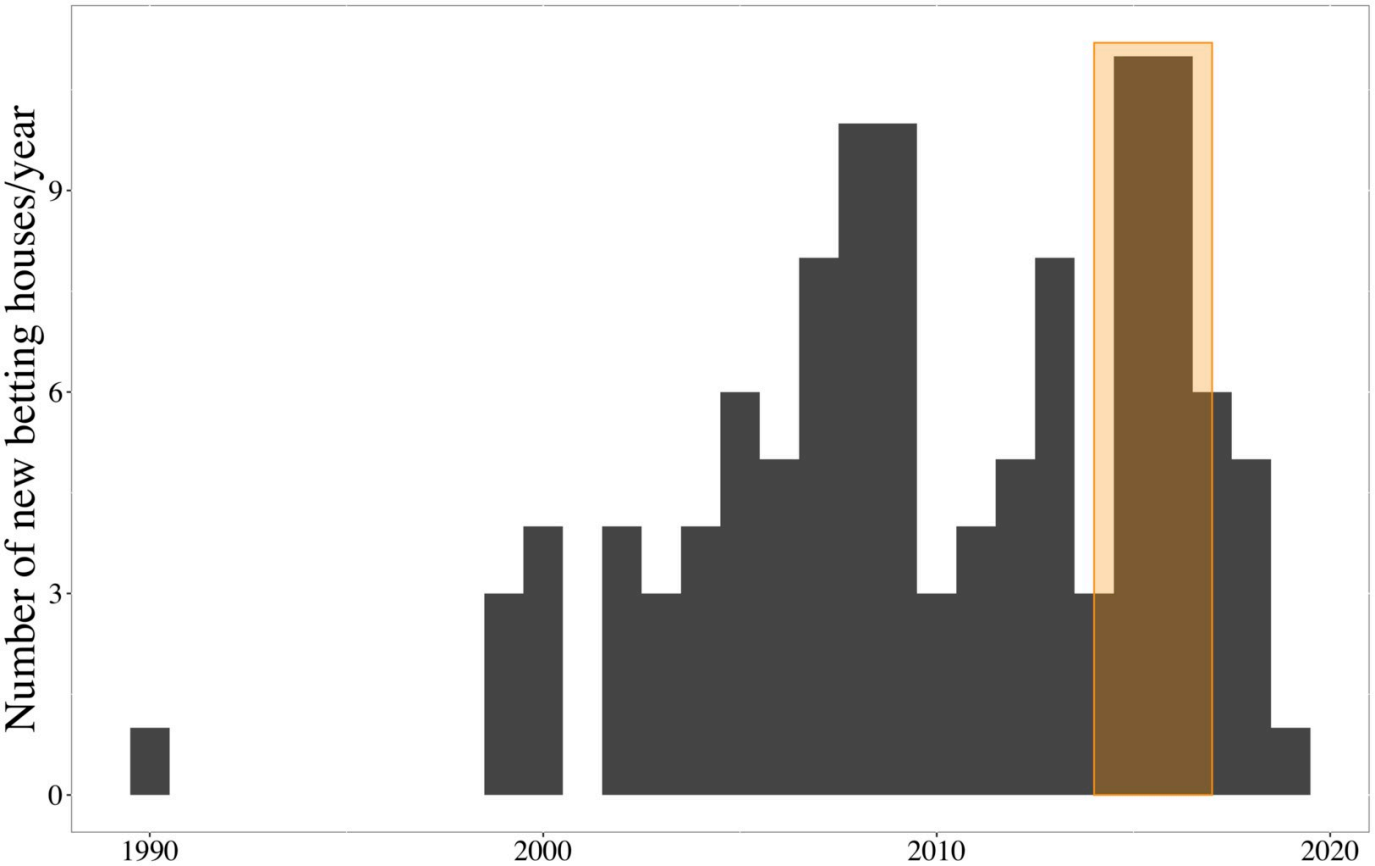
Betting houses openings. Opening year of ongoing betting houses in Madrid from 1990 to 2019. Note: Census data gathered from the Madrid City Council open data portal.

This study proposes a solution to these limitations. First, we compare the evolution in academic performance of geographically close and similar high schools, which differed in one thing—one of them was recently exposed to a new betting house while the other was not. Secondly, using a case where vulnerable populations were not more likely to be exposed to betting houses. We do so by looking at the case of Madrid (Spain), where rich available data enables us to estimate the effect of gambling opportunities on educational performance in less wealthy areas (public high schools) and neighborhoods with a higher income level (charter high schools). This choice is also motivated by the particularly intensive spread of new betting houses experienced in Madrid between 2015 and 2017—see Fig 1, the orange-underscored area.

Spain decriminalized gambling during the first post-authoritarian administration, in 1977 (Decree 16/1977) but did not regulate the gambling industry until the 1980s—the first regulatory attempt coming from the region of Catalonia in 1984, followed by Galicia in 1985 [32]. Madrid passed its first gambling law in 2001, which became outdated after the internet revolution of the 2000s. For this reason, the Spanish parliament passed the first national-level law regulating gambling in 2011. Nevertheless, the somewhat vague Spanish Law 13/2011 was far from closing the debate about gambling companies’ taxation and its potential negative externalities. National authorities promoted this lacking legal definition to decentralize the responsibility (and the benefits) from gambling regulations to regional authorities. For instance, the national law specified that minors were banned from gambling facilities, but left the responsibility to regions to detail any limitation for new betting houses’ distance to high schools, hospitals, or civic centers. Surprisingly enough, the first regional gambling regulation updates were passed between 2018–2019, leaving a decade to expand and normalize such a business sector.

In any case, previous and current legal cross-regional differences resulted in variations in the restrictions imposed on gambling companies and protections offered to vulnerable groups, which partly account for the contrasting development of this sector in Madrid or Murcia compared to Barcelona or Valencia. Madrid authorities’ minor reaction to the initial spread of gambling facilities and its negative externalities allowed for short-term expansion. The government of the Comunitat Valenciana, for instance, legally restricted new betting shops openings at more than 850m from the educational center and closed existing betting houses at less than 500m from public facilities (Law DOGV no. 8834 passed on June 15th, 2020). In contrast, while Madrid’s reform also includes ID controls at betting houses’ entrances and imposes 9,000€ penalties, it only restricts *new* gambling licenses to requests located at more than 100m. from the closest educational center (Decree 42/2019 of Madrid’s region). Focusing on the case of Madrid, its first betting house opened in 1990, but 119 Most of these betting shops are devoted to sports betting, but also include other games of chance such as slot machine and electronic gaming machines. More have opened since then—a growth of stores that is far from linear. After the legalization of sports betting in 2006, fifteen betting houses opened their doors in Madrid before 2011. Nevertheless, the spread of gambling facilities peaked in 2015–2017, when more than twenty-five betting houses opened in the capital city. This expansion increased Madrid citizens’ exposure and accessibility to gambling facilities, and regardless of the ban on underage gambling, also its popularity among teenagers: adolescents’ gambling prevalence increased by 30% in Spain between 2016 and 2019 [2, 33]. The ESTUDES report exalts that the share of adolescents that gambled during last year rose from 21% in 2016, to 30% in 2019 [2, 33]. According to ESPAD’s estimation, 10% of Spanish adolescents gambled during 2019 [34].

This spread of betting shops, affecting socio-economically diverse areas, turned Madrid into an instrumental case to examine the (potentially heterogeneous) consequences of gambling on human capital formation. We leverage the shock of exposure to close-by new gambling facilities suffered by some high school students to assess its consequences on academic performance. Understanding high schools as aggregate units of individual-level behavior, which respond to contextual factors such as the supply of gambling facilities, we draw on administrative data to measure high schools’ educational performance. A more detailed explanation of the data and its sources can be found in the S1 Text. It is worth noticing that the Spanish educational system comprises three levels: compulsory education (primary and lower-secondary education), higher secondary education (academic and vocational training), and tertiary education. We selected high schools that offered the Academic Track (N = 277). This training lasts for two years and gives access to tertiary education after passing a standardized state-level exam. Therefore, our population of analysis is a selected share of high school students that seek to access university and, in most cases, are between sixteen and nineteen years old. S2 Fig the geographic distribution of betting houses (in red crosses), and high schools (public, in blue, and charter, in red circles) in Madrid by 2017.

Building upon extant literature exalting the role of gambling facilities’ availability or supply as a predictor of its consumption during adulthood and adolescence [34–38], we estimate the effect of exposure to new betting houses on high school educational performance using a difference-in-differences setup (DiD). In our study, the DiD estimator compares the evolution of high schools’ performance before and after some schools became exposed to new betting houses between 2015 and 2017. This design hinges on the assumption that treated and non-treated high schools followed a similar educational performance trend before the betting houses opened—also known as the parallel trends assumption. We provide evidence supporting the plausibility of this claim. Building on that, the differential evolution in the educational performance of high schools suddenly exposed to betting houses, compared to the trend followed by schools not exposed to such stores, can be attributed to the independent effect of an increase in the supply of gambling facilities.

We find that betting houses unevenly harm average grades of nearby high schools in state-level exams. This harm is only present among public high schools in low-income areas. Compared to other public high schools situated in low-income areas, we find that those high schools located less than 500m from a new betting house decrease their average grade by 0.6 points on a 0–10 scale (the average mark is 6.1). In line with the Compensatory Advantage theory, we do not find such an effect on charter schools or public high schools located in neighborhoods above the average income level [39]. According to this theory, we expect that students living in high-income areas or attending charter schools have a security net that prevents them from falling into certain behaviors such as gambling. Furthermore, we expect that gambling’s adverse effects are less detrimental for children from more advantageous families.

This study provides novel empirical evidence of the negative consequences of gambling on one outcome affecting human capital formation. Only the most vulnerable collectives suffered from the negative consequences of an increase in Madrid’s gambling supply. Unfortunately, the conditions for these results to hold—an increase in the supply of betting houses around vulnerable populations—are met in many Spanish and worldwide cities beyond Madrid. Hence, these findings have relevant policy implications for the ongoing debate on the regulation of gambling and betting houses. By filling the gap of measuring the effect of gambling on adolescents’ human capital formation process, this paper sets the basis for guiding and supporting the state or supranational-level regulation of betting houses, which, according to our results, are undermining equality of opportunities.

## Materials and methods

We measure high schools’ educational performance using administrative data detailing the average grade obtained by each school at the standardized state-level exams that give access to the university. These state-level exams are comparable to, for example, the A-level exams in the United Kingdom or the SAT in the US. This exam is equal for all students in the whole region of Madrid, and it is only taken by those students that (1) successfully manage to finish the academic track and (2) wish to access university. We acknowledge that test performance is a function of both knowledge and motivation. Given that this standardized state-level exam is a “high stakes” exam, we ensure that we are taking into account “test-taking motivations”, avoiding concerns as to whether grades collected are (or not) a valid measurement of students’ performance [40]. Moreover, these exams are anonymous and marked by external evaluators, which ensures that teachers within schools are not *grading on a relative curve* according to average classroom performance [41]. Accessible data on outcomes restrict the temporal scope of the study to 2014–2017. Indeed, the authors only access aggregate data by neighborhood and high schools—i.e., no individual data was used. Moreover, being betting houses placement independent to high school’s grade evolution, one may consider that as a natural experiment in which we, as researchers, cannot control the treatment administration.

### Identification strategy

We seek to identify should students’ performance decrease by opening a betting house from one course to the following one. This short-term effect permits us to rule out the existence of a compositional effect—i.e., students self-selecting into high schools farther away from betting houses. As the academic track lasts for two years, students start this program before public authorities issued the license for a betting house that they would be exposed to during their test year. To support this claim’s robustness, we observe a placebo outcome: the number of students that sit for the state-level exam.

The 2015–17 wave of new betting house openings in Madrid is a suitable case as it provides twofold motives for isolating gamblers’ selection from its consequences. First, prior research associates increase in gambling availability (supply) with the growth of gambling participation and its associated pathologies (demand) [34–38]. Building on these papers and prior reports stating the prevalence of problem gambling among adolescents, we expect an increase in gambling facilities will lead to a growth of problem gambling in those areas.

Second, comparing public (89 high schools) with charter high schools (188 high schools) would be imprecise given that the two types of schools have different schedules. While most public high school students attend classes from 8:00 to 14:30, students in a charter or private high school also have lessons during the afternoons. Furthermore, unlike public schools, charter schools are characterized by their extensive supply of extra-curricular activities right after the afternoon lessons. Therefore, exposure to leisure activities outside school is lower and more supervised among high school students attending charter high schools. For this reason, although public and charter schools are equally likely to be close to a betting house (see S4 Table), we expect an uneven effect of betting houses across different types of schools. An increase in the gambling supply is likely not to imply an effective increase in the gambling demand among charter or private school students. Unfortunately, we do not have survey data to test such a claim. Indeed, this argument applies to public schools located in high-income areas where social models of leisure, commuting patterns, and a more monitoring parental style might prevent adolescents from bad habits like gambling [18, 42, 43]. To avoid comparing apples with oranges, this paper solves the other part of the selection problem by matching high schools by type and neighborhood income level.

### Exposure to gambling

Using a difference-in-differences setup, we leverage the variation in high schools’ exposure to gambling facilities as the treatment. We use two different specifications capturing exposure to such treatment. First, we use individual-level survey data on commuting patterns among Madrid citizens to uncover that the average students’ distance to educational centers is 500m. See S1 Table We employ this radius to divide Madrid high schools using a dummy variable switching on those education centers exposed to a betting house at less than 500m. S4 Fig plots the evolution of treated and non-treated high schools’ density according to this criterion. Second, we employ the high school/year logged meters distance to its closest betting house as a continuous measure of exposure to gambling facilities. We utilize this specification because exposure may drop much precipitously at shorter than longer distances, where it matters less. The log transformation accordingly weights variations in small distances more than in higher numbers. S4 Table details the evolution of high schools’ average distance to the closest betting house. As expected, given the wave of new openings experienced between 2015 and 2017, high schools’ average distance to betting houses decreases by 10% during this period.

### Estimation

We employ a Difference-in-Differences set up to exploit such increasing exposure to betting houses and estimate its effect on educational performance. For that purpose, we use the following two-way fixed effects (TWFE) equation:

$$\begin{array}{c} Y_{it}=\alpha +\gamma_{i}+\lambda_{t}+\tau D_{it}+u_{it} \end{array}$$ (1)

where *Y*_*it*_ is the dependent variable, high school *i*’s academic performance at time period *t*; *γ*_*i*_ is a high school fixed effect that controls for unobserved heterogeneity; λ_*t*_ is a time-period fixed effect that controls for common shocks in all high schools’ academic performance; and *τ* yields the treatment effect of having a new betting house at less than 500m. We replicate this model using the continuous variable capturing exposure to the betting houses, where *τ* lends the average effect for increasing high schools distance to the closest betting house in 1 logged meter.

Our study is a paradigmatic example of staggered DiD—different units adopt the treatment status at different time-periods and remain exposed at least until the last time-period (see S4 Fig for illustration). In those cases, TWFE models may wrongly categorize treated observations at the control group due to not changing treatment status in *t*_+1_ or *t*_+2_ periods after being treated since *t*_0_. As a result, the estimand of standard two-way fixed effects models incorporates a bias, a weight that might offset the real treatment effect. We attempt to rule out this bias using the group-time average treatment effect estimator proposed by Callaway and Sant’Anna [44]. This methodology calculates and discounts this bias, controls potential heterogeneity in the treatment effect across time-periods and subgroups, and helps estimate reliable pre-treatment trends.

**Fig 2:**
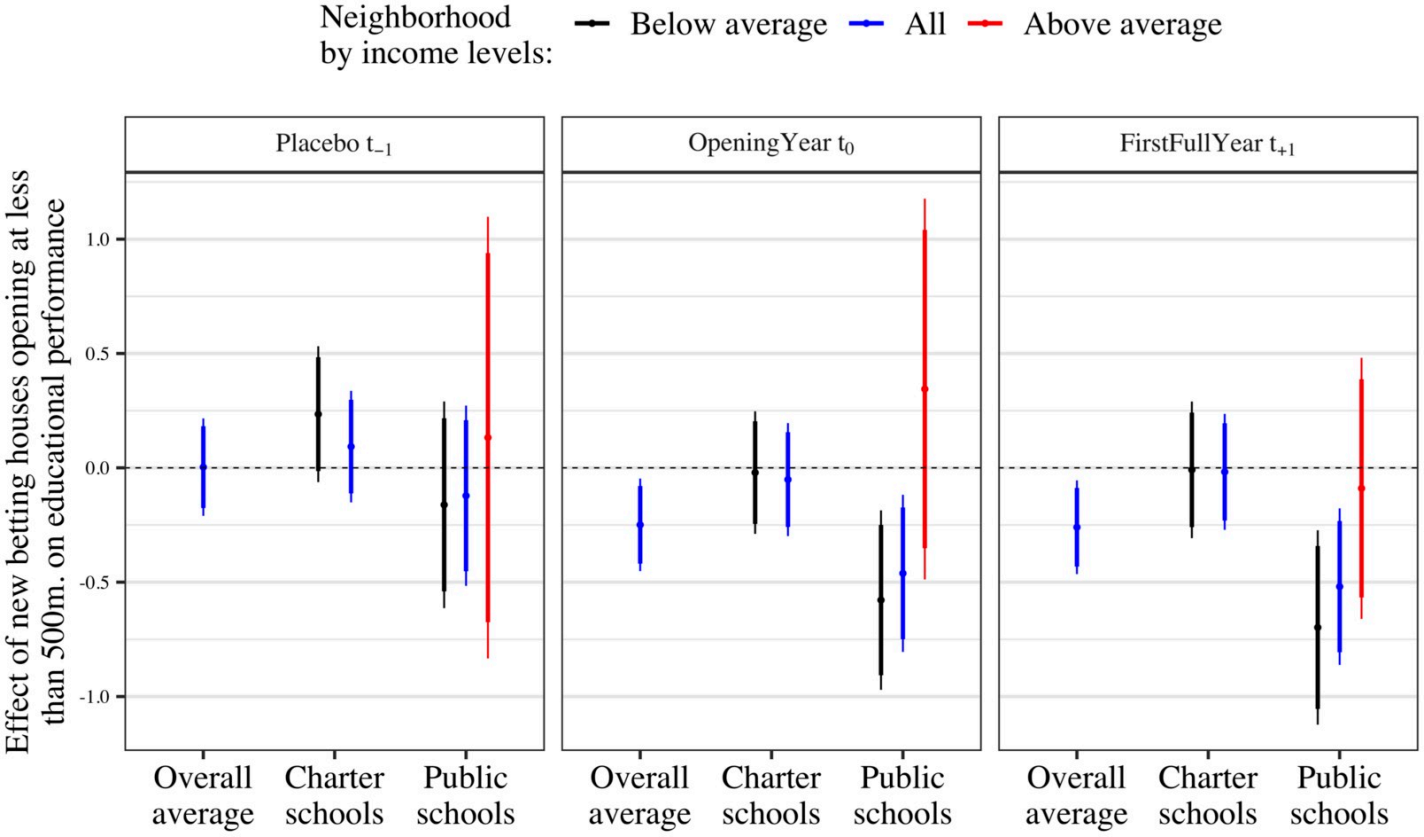
Main result. The estimated effect of exposure to betting houses on educational performance using TWFE estimators. Note: The figure shows the point estimates and robust 95% (thin) and 90% (thick) confidence intervals estimated using Eq 1. All models include school-level and year-fixed effects. The outcome variable is the school-level average grade obtained by all the students that sit the state-level exam (mean, 6.26; SD, 0.7). The overall sample contains 235 high schools, 149 charter, and 86 public. Models on the effect of betting houses on charter schools in neighborhoods below income average use 75 high schools, and those in neighborhoods above the average income 75 schools. Models focusing on the effect on public high schools include 62 high schools (242 observations) when analyzing below-average-income areas and 25 schools (100 observations) when approaching the effect of gambling facilities on public high schools located in high-income neighborhoods of Madrid.

Indeed, the use of a Difference-in-Differences setup for assessing the effect of betting houses on academic performance may hinge on the parallel trends assumption. For control units to credibly represent a post-treatment benchmark for treated units in the absence of treatment, both groups should follow similar change rates before one group adopts the treatment. The results plotted in the first panel of Fig 2 and *t*_−1_ and *t*_−2_ of Fig 4, obtained using TWFE and Sant’Anna and Callaway’s estimators, respectively, suggest that parallel trends assumption is likely to hold in this case study. The overall average treatment effect (blue spike, first column in Fig 2) presents a precisely estimated zero-effect or null finding (b = 0.003, s.e. = 0.109, p = 0.978). In other words, gambling companies did not target areas or high schools with worsening academic performance.

## Results

Fig 2 shows the estimates for the treatment effect obtained employing Eq 1 (Eq 1). This figure comprises three panels. Each panel presents the estimated treatment effect in every time-period: before betting houses opened (placebo), during the opening year, and the full year after the opening occurred. Moreover, each panel includes different specifications. Columns split the sample by types of high schools. The first column presents the results obtained using all high schools in Madrid, the overall average treatment effect. The second column compares treated to non-treated charter high schools. The third column shows the estimated effect only using public high schools. Each spike includes the point estimate and the 90% and 95% confidence intervals estimated when only using schools located in neighborhoods above (in red) or below (in black) Madrid’s average neighborhood average income level. Blue spikes plot the results of models using high schools located in both wealthier and more deprived areas (See Data description section in the S1 Text further details). There is not enough variation in the exposure to betting houses among charter high schools located in above-average to estimate its effect.

### Main results

Fig 2’s central panel exhibits the main finding of this paper: the short-term effect of new betting house openings. The central panel’s first column shows the average treatment effect of betting houses: high schools’ performance declines by 0.25 points on a 0 to 10 scale when a betting house opens at less than 500m (b = -0.249, s.e. = 0.103, p<0.05). The second and third columns compare betting houses’ effect on charter (left) and public schools (right).

In line with our expectations, both point estimates in the second column indicate that betting houses had no immediate effect on charter schools’ performance. In contrast, betting house openings close to public high schools located in neighborhoods below the average income level decreased their average grade by 0.5 points (1 standard deviation, p<0.05) on a 0–10 scale, being more robust in public schools located in low-income neighborhoods (b = -0.578, s.e. = 0.198, p<0.05). We estimate the differential effect of novel exposure to betting houses on the public with respect to charter high schools by interacting the treatment with a dummy variable representing public high schools. S15 Fig presents the interaction term when using all schools and only those located in neighborhoods below the avg. income. The differential impact of betting houses in public compared to charter schools is of 0.4 points in a 0–10 scale.

The third panel presents the estimated effect for *t*_+1_, the first full academic course after the betting house opened. Results essentially confirm and aggravate the short-term consequences of increasing the supply of gambling facilities. While charter schools remain entirely unaffected, betting houses occasion a 0.7 decline in poorer, public high schools academic performance (b = -0.697, s.e. = 0.215, p<0.01).

**Fig 3:**
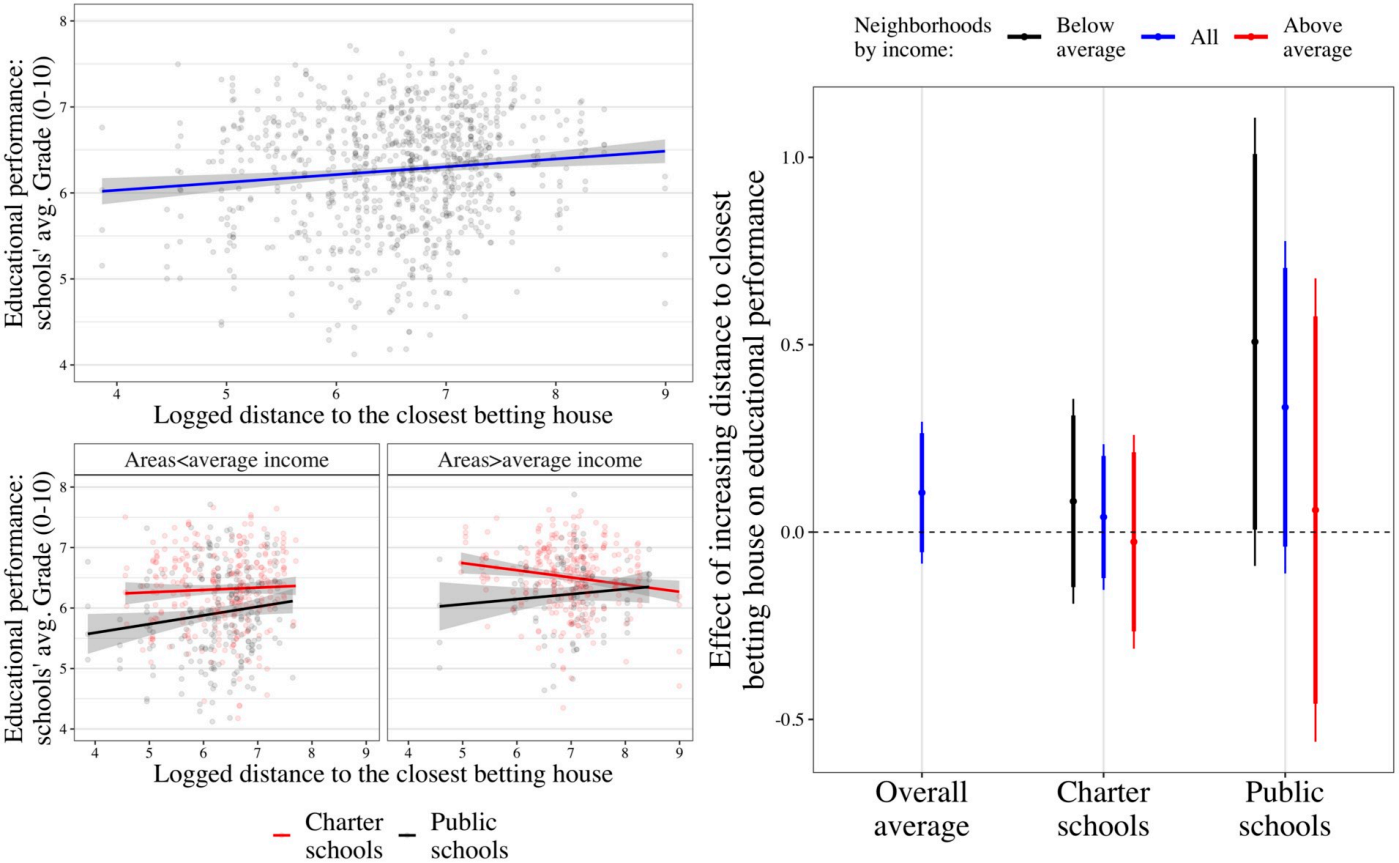
Estimated effect of distance betting houses on educational performance using TWFE estimators. Note: The figure includes three panels. The top-left panel shows the linear association between distance and academic performance. The bottom-left splits the top-left panel into four subgroups by neighborhoods’ income level and type of school. The right-hand side panel shows the TWFE models’ results (following Eq 1) but using a continuous variable of exposure to betting houses—logged meters distance.

To further test our results, we also used distance as a continuous variable. The three panels in Fig 3 presents the association between high schools’ distance to betting houses and its academic performance. The two plots on the left side display descriptive figures. At the top-left corner of the Fig 3, the first panel plots the association between high schools logged meters distance to the closest betting house (horizontal axis) and their average grade obtained at the state-level exam (vertical axis) using all high schools in Madrid between 2014 and 2017. The blue line represents the linear regression estimation, and the shaded area is the 95% confidence interval. Notice that smaller values in the horizontal axis (less distance to the closest betting house) capture higher exposure to gambling facilities, which should accordingly relate to lower educational performance.

In this line, the linear prediction shows a positive association between distance to the closest betting house and academic success. The two panels below split the sample into four groups. While the left-side panel uses high schools located in neighborhoods below Madrid’s median income level, the right-side only uses high schools in richer areas. In each panel, high schools and the linear predicted effect of distance on grades are, in turn, divided by the type of high school: charter (in red) and public (in black) high schools.

In line with prior results, the negative association between exposure to betting houses and academic performance is mostly attributed to the most vulnerable groups—public high schools in poorer areas. This descriptive intuition is confirmed when replicating the difference-in-differences setup but using the continuous variable as the treatment. Results are shown in Fig 3 right side panel. The average short-term treatment effect of increasing distance to the closest betting house (first column) is positive but not statistically significant at any level of confidence. Distance to betting houses only shows a confidently positive and robust association with academic performance when restricting the sample to the most vulnerable populations: public high schools in poorer neighborhoods (third column, black spike; b = 0.508, s.e. = 0.303, p<0.10). Acknowledging the non-linear distribution of logs, the bottom line of these results is that decreasing vulnerable high schools’ distance to the closest betting house by 300m. decreases their academic performance by 0.5 points on a 0–10 scale.

All in all, DiD and TWFE estimators reflect that betting houses harm academic performance in public high schools located in low-income areas of Madrid. Indeed, new betting houses do not harm the academic performance of close-by charter schools. This finding can be attributed to the expected uneven effect of new betting houses. As predicted by the Compensatory Advantage theory, charter schools might prevent students from gambling by including alternative activities such as afternoon lectures and extra-curricular activities. That said, many family-level factors that in turn affect selection into schools might be the moderators causing this missing association—i.e., different social models of leisure, commuting patterns, and a more monitoring parental style [18, 42, 43].

### Robustness checks

This section presents four different tests supporting the robustness of the main findings. The application of the Callaway-Sant’Anna estimator to correct for potential biases resulting from two-way fixed effects models in settings with dynamic adoption of the treatment; the consistency of exposure distinguishing different intensity levels—i.e., monotonicity; placebo tests using Starbucks; and finally, examining the plausibility of compositional changes as an alternative explanation using the number of students sitting in for the exam.

**Fig 4:**
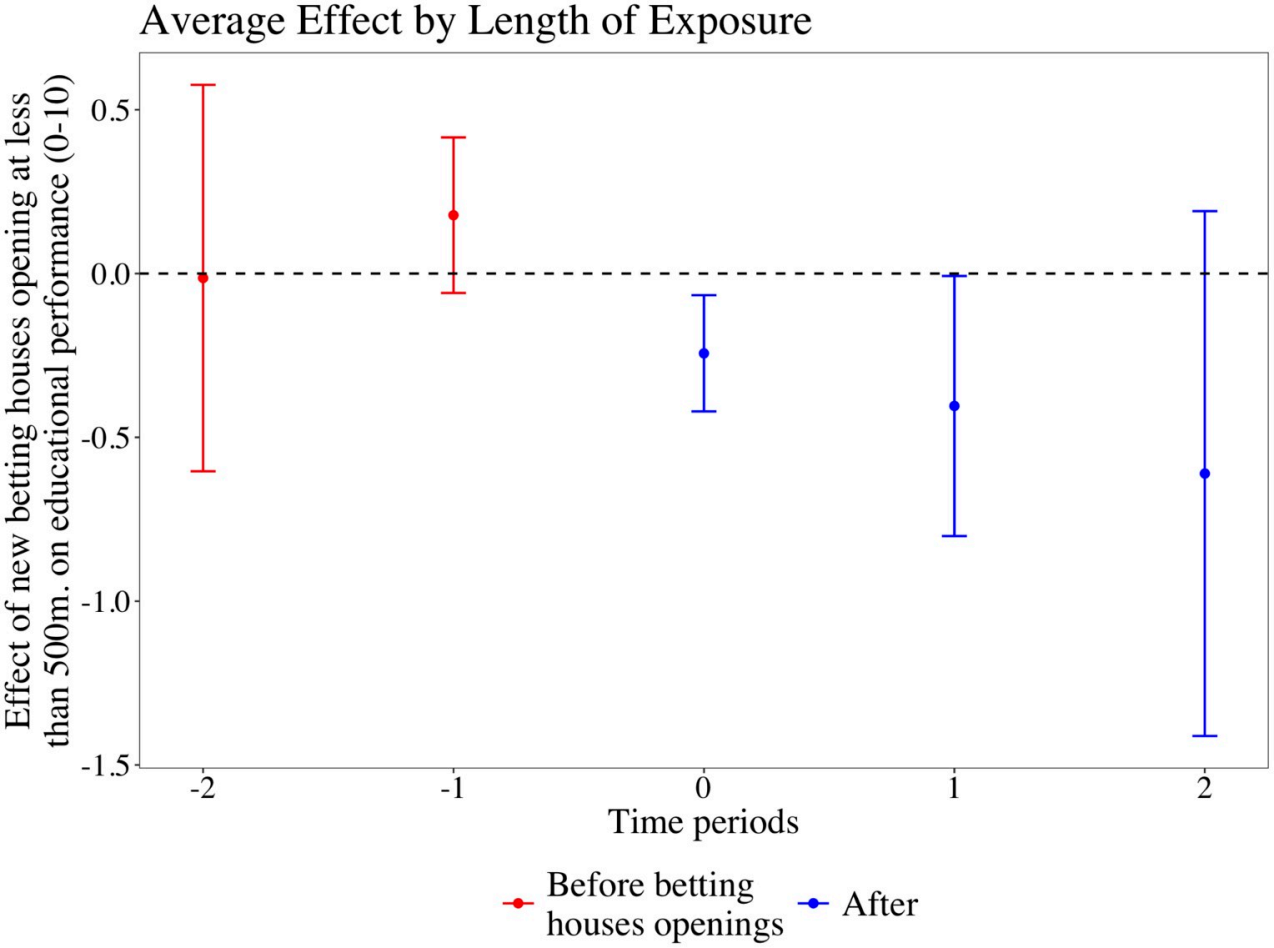
Estimated effect of distance betting houses on educational performance using the Callaway-Sant’Anna estimator. Note: Effect of proximity to betting houses on high school’s average mark in state-level exams. Point estimates and Confidence Intervals are obtained using a regression model that allows for effects before and after betting houses were opened.

The Callaway-Sant’Anna estimator adapts TWFE estimators to settings with staggered treatment administration by discounting treated observations from the control group from *t*_+1_ onwards [44]. Fig 4 presents the output from using the group average treatment effect estimator proposed by these authors. The plot illustrates the estimated difference between high schools with a close-by betting house in every period—before (in red) and after public authorities issue licenses (in blue). Conclusions from previous analyses remain intact.

Suppose the argument made in this paper is valid. In that case, we should observe public high schools located closer than 500m., for instance, 200m., to show a more reliable and substantial academic decline. We test this hypothesis by dividing the treatment dummy into two: one for high schools at less than 500m. and another at less than 200m. S19 Table suggests that regardless the low number of observations falling in this category, the decrease in academic performance is slightly more pronounced in public high schools at less than 200m of new betting houses.

An alternative explanation for such a finding is that any new leisure setting may hinder high schools’ performance by distracting students. As prior research exalts, this is not the case, and betting houses may be particularly harmful, especially for vulnerable populations. We seek to confirm this expectation by replicating our DiD setup employing the variation generated by new Starbucks cafes in Madrid. S11 and S12 Figs Placebo test: Starbucks section presents the results. We find no evidence that Starbucks affected academic performance in either poorer nor richer high schools.

**Fig 5:**
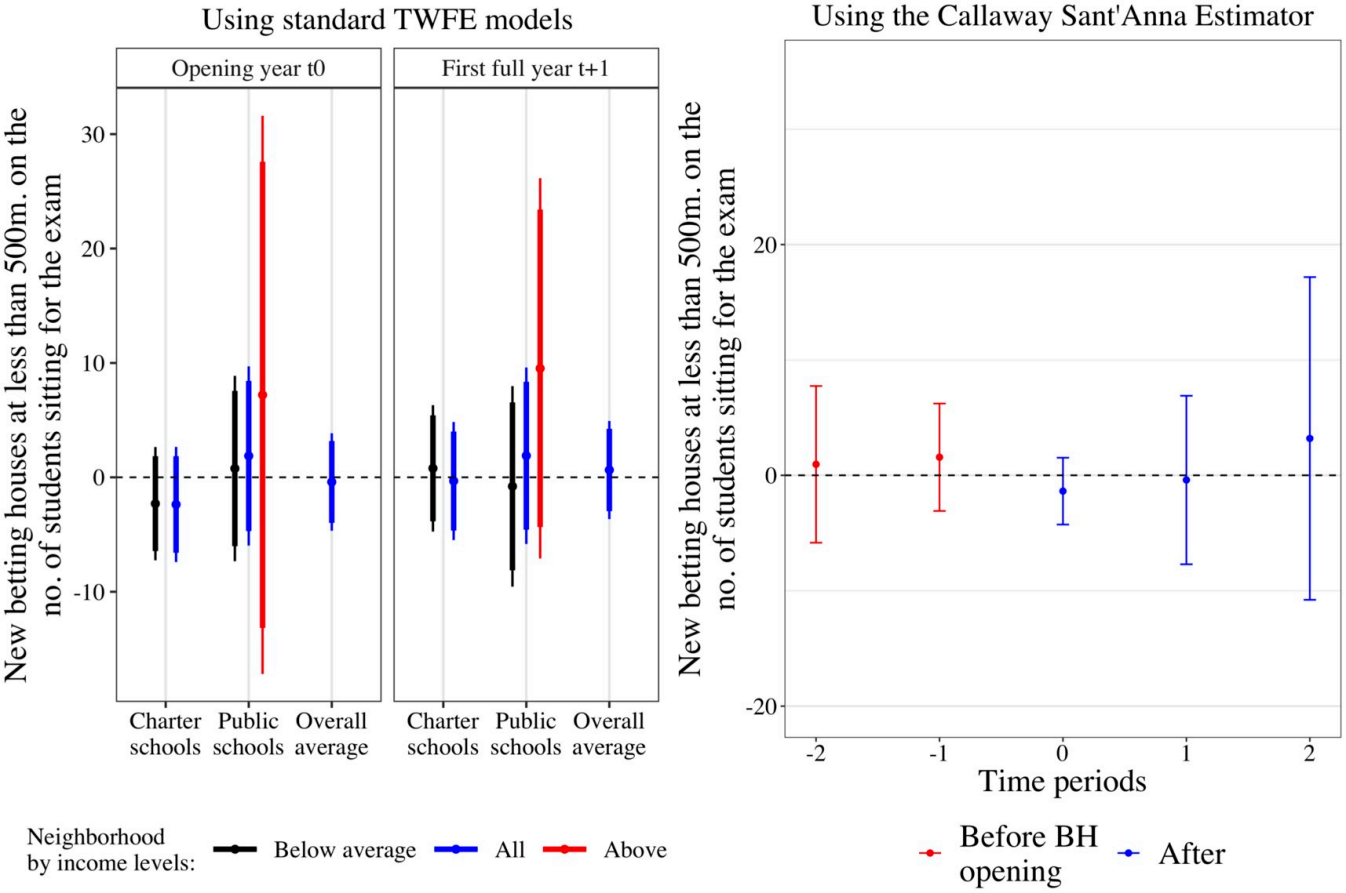
Alternative explanation: Compositional change. Betting houses effect on the no. of students. Note: The figure shows a replication analysis of Figs 2 and 4 but replacing this paper’s primary outcome with the number of students sitting for the exam. Notice that an adverse effect would inform that students sort out from high schools with close-by betting houses—the negative effect on academic performance would come from a compositional change.

Another alternative explanation is that our results could be driven by changes in the composition of students and neighborhoods. For example, a potential mechanism explaining the decline in high school’s academic performance is that their most outstanding students might sort themselves into other high schools farther away from in-degradation contexts such as the one given by betting houses. Should betting houses affect educational performance but not the composition of students who take the exam, we must find the number of students who sit in for the state-level exam unrelated to having a new betting house nearby. Fig 5 shows that the association between new betting houses and the number of students is not only positive but small and indistinguishable from zero, suggesting that there is no sorting driven by betting houses. One might also think that betting houses may devalue neighborhoods in the short run, decreasing students’ academic performance. S10 Fig replicates our DiD setup using avg. parish rent price. We find no evidence that gambling companies target already impoverishing areas to open betting houses, nor that betting houses devalue an area’s rental prices in the short run.

## Discussion

This study examines the impact of sports betting houses on the educational performance of high schools. We exploit the quasi-random variation yield by high schools’ proximity to new betting houses opened in Madrid to assess whether an increase in gambling facilities’ supply affects adolescents’ performance at state-level exams. Classifying high schools by type and income level allows us to isolate the moderation or selection effect grasped by income on the impact of betting houses on educational performance. Using a Difference-in-Differences estimator, we found that, in Madrid, public high schools located in low-income areas decrease their educational performance when a new betting house opens at less than 500m. We conversely find no such effect when looking at charter high schools or other public schools located in areas above the average Madrid’s income level.

As stated above, the effects of gambling are not small nor diminishing with time. They represent meaningful changes in school performance, which generated greater inequality. Betting houses’ effect is almost double the baseline difference between public and charter schools. This confirms that gambling is most detrimental for vulnerable populations.

It is worth noticing that these effects occur despite our reliance on a positively selected share of students. First, these students have surpassed the first critical educational transition in Spain (from compulsory to post-secondary education), and most will pursue a university degree. The Spanish educational system is characterized by a mismatch between higher numbers of people with either primary or tertiary education and an insufficient population with upper secondary education [45]. Hence, most students who manage to finish Compulsory Education and enroll in higher secondary education acquire tertiary education. Second, the academic track is considered the most prestigious and demanding track within post-compulsory education. Therefore, these students are positively selected for their educational aspirations and motivations compared to students of the same age who attend vocational training or have compulsory education. That said, these findings are not exempt from limitations and assumptions. This paper essentially employs the reduced version of an instrumental design. In other words, we build on previous evidence supporting that gambling accessibility (Z), its density and distance, increases gambling consumption and problem gambling (X) [16, 30] to assess the consequences of gambling accessibility (Z) on human capital formation (Y). However, there is no data actually to validate the instrument in our case study. Further research should attempt to fill this gap using individual or student-level data.

How do these results translate to other contexts? We expect these results to hold all over Spain, especially in big cities such as province capitals, as both scope conditions are met: vulnerable populations—public high schools in low-income areas, and handy betting houses. One of the most popular gambling companies maps all their more than 1K on-site betting houses in Spain Source: https://m.apuestas.codere.es/csbgonline/home/mapCond?=mad—Visited April 1st, 2021, and regions like Murcia, Andalucía, or Galicia seem as affected as Madrid. These scope conditions are unfortunately all too often found outside Spain as well. Italy, the UK, some US states, and other OECD countries such as Sweden are starting to recognize gambling as a public health issue [46]. In some of these countries, adolescents are one of the most affected collectives. For instance, in Italy, where betting houses are legal, more than 4% of 15–19 years old are considered “problem gamblers” with an addiction [47], and 37% of 11–16 year-olds in England and Scotland gambled in 2018 [48]. In fact, by the age of 14, more than 11% of children in the UK have gambled. Authors’ own calculations using wave 6 of The Millennium Cohort Study, which is a longitudinal study following 16,000 children born around the 2000s in England, Scotland, Northern Ireland and Wales. In Croatia, similar to many other countries in the Balkans, about 19.4% of high school students regularly bet on sports [26], and between 8 to 12% of high school students in Zagreb display risky gambling patterns [49]. All in all, evidence suggests that countries and cities which turned into a legislative liberalization of gambling and betting, such as Zagreb or Madrid, suffered from a sudden escalation of betting shops, in particular, sports betting, which increased gambling opportunities in these cities. As pointed by Ricijas [26], even though gambling is an activity legally intended only for adults, youth throughout Europe have access to some sort of game of chance before they turn 18 [18, 50, 51]. Beyond gambling availability, current regulations are also failing at protecting our youth from accessing gambling facilities.

These findings have critical implications for designing policies tackling the increase of unequal opportunities promoted by betting houses. Some Italian regions limit new betting houses to a minimum of 500m distance from schools. In Spain, several regions, like Murcia or Aragón, passed laws limiting the minimum distance between a betting shop and an educational center. Spain’s current debate proposes different limits for the distance from betting houses to schools, with proposed levels between 100m. and 500m. These findings back those on the most conservative side: strong abutments should be employed to prevent vulnerable populations from falling into addictive dynamics. That said, this work performs a conservative school-level estimation of these effects using Madrid’s case study, and further work is then needed to delve into the dynamics and mechanisms through which gambling supply diminishes educational performance. Examining the consequences of existing differences in advertising politics such as whistle-to-whistle or shirt sponsoring bans between Italian and British regions, as well as the (lack of) implementation of legal age for gambling could help us understand the mechanisms and help us accurately design policies approaching the spread of new addictions among young generations.

## Supporting information

S1 Table Madrid underage commuting summary statistics. Note: Authors own elaboration. Data source: The 2018 Household Mobility Survey conducted by the Consorcio de Mobilidad de Madrid.
(TEX)

S2 Table Summary statistics. Note: Data obtained from the Madrid City Council’s census and the education authorities of the Region of Madrid. The authors’ estimated high schools-betting houses yearly distances.
(TEX)

S3 Table High schools’ distance to the closest betting house. Note: Authors’ own elaboration. Data obtained from the Madrid City Council’s census and the education authorities of the Region of Madrid. The authors’ estimated high schools-betting houses yearly distances.
(TEX)

S4 Table High schools’ likelihood of being exposed to betting houses at less than 500m. Comparison of public and charter schools. Note: Authors’ own elaboration. Data employed originally comes from the Madrid City Council’s census and the education authorities of the Region of Madrid. The authors’ estimated high schools-betting houses yearly distances.
(TEX)

S5 Table Exposure to close-by betting houses on High schools’ average performance. This table includes the pre-openings placebos and the actual average treatment effect. Note: This table includes the pre-openings placebos and the actual average treatment effect. Authors’ own elaboration. Data employed originally comes from the Madrid City Council’s census and the education authorities of the Region of Madrid. The authors’ estimated high schools-betting houses yearly distances.
(TEX)

S6 Table Effect of betting houses when setting the treatment one year after opening, in *t*_+1_. This table distinguishes schools by type and income level. Note: This table distinguishes schools by type and income level. Authors’ own elaboration. Data employed originally comes from the Madrid City Council’s census and the education authorities of the Region of Madrid. The authors’ estimated high schools-betting houses yearly distances.
(TEX)

S7 Table Effect of betting houses when setting the treatment in the BH opening year, in *t*_0_. This table distinguishes schools by type and income level. Note: This table distinguishes schools by type and income level. Authors’ own elaboration. Data employed originally comes from the Madrid City Council’s census and the education authorities of the Region of Madrid. The authors’ estimated high schools-betting houses yearly distances.
(TEX)

S8 Table Placebo test. Effect of betting houses before BH’s opening year, in *t*_−1_. This table distinguishes schools by type and income level. Note: This table distinguishes schools by type and income level. Authors’ own elaboration. Data employed originally comes from the Madrid City Council’s census and the education authorities of the Region of Madrid. The authors’ estimated high schools-betting houses yearly distances.
(TEX)

S9 Table Placebo test. Effect of betting houses two years before BH’s opening year, in *t*_−2_. This table distinguishes schools by type and income level. Note: This table distinguishes schools by type and income level. Authors’ own elaboration. Data employed originally comes from the Madrid City Council’s census and the education authorities of the Region of Madrid. The authors’ estimated high schools-betting houses yearly distances.
(TEX)

S10 Table TWFE models estimated effect of (logged) distance to the closest betting house on educational achievement. This table distinguishes schools by type and income level. Note: This table distinguishes schools by type and income level. Authors’ own elaboration. Data employed originally comes from the Madrid City Council’s census and the education authorities of the Region of Madrid. The authors’ estimated high schools-betting houses yearly logged distances.
(TEX)

S11 Table Compositional change, alternative explanation: Sorting. Betting houses on the number of students sitting in for the exam. Note: Authors’ own elaboration. Data employed originally comes from the Madrid City Council’s census and the education authorities of the Region of Madrid.
(TEX)

S12 Table Compositional change, alternative explanation: Betting houses on the no. of students one year after BH opening, in *t*_+1_. This table distinguishes schools by type and income level. Note: This table distinguishes schools by type and income level. Authors’ own elaboration. Data employed originally comes from the Madrid City Council’s census and the education authorities of the Region of Madrid.
(TEX)

S13 Table Compositional change, alternative explanation. Betting houses on the no. of students one year after BH opening, in *t*_0_. This table distinguishes schools by type and income level. Note: This table distinguishes schools by type and income level. Authors’ own elaboration. Data employed originally comes from the Madrid City Council’s census and the education authorities of the Region of Madrid.
(TEX)

S14 Table Compositional change, alternative explanation: Betting houses on the no. of students one year after BH opening, in *t*_−1_. This table distinguishes schools by type and income level. Note: This table distinguishes schools by type and income level. Authors’ own elaboration. Data employed originally comes from the Madrid City Council’s census and the education authorities of the Region of Madrid.
(TEX)

S15 Table Compositional change. Betting houses on district rent prices in euros/m2, in *t*_+1_. This table distinguishes schools by type and income level. Note: Authors extracted the data on rent prices extracted from Madrid open access records, “Renta mensual de la vivienda en alquiler (€/m2 construido) por Distrito y por Trimestre”. Note: Authors’ own elaboration. Data employed originally comes from the Madrid City Council’s census and the education authorities of the Region of Madrid. The authors also extracted the data on rent prices from Madrid open access records, “Renta mensual de la vivienda en alquiler (€/m2 construido) por Distrito y por Trimestre”.
(TEX)

S16 Table Compositional change. Betting houses on district rent prices in euros/m2, in *t*_0_. Note: This table distinguishes schools by type and income level. Authors’ own elaboration. Data employed originally comes from the Madrid City Council’s census and the education authorities of the Region of Madrid. The authors also extracted the data on rent prices from Madrid open access records, “Renta mensual de la vivienda en alquiler (€/m2 construido) por Distrito y por Trimestre”.
(TEX)

S17 Table Compositional change. Betting houses on district rent prices in euros/m2, in *t*_−1_. Note: This table distinguishes schools by type and income level. Authors’ own elaboration. Data employed originally comes from the Madrid City Council’s census and the education authorities of the Region of Madrid. The authors also extracted the data on rent prices from Madrid open access records, “Renta mensual de la vivienda en alquiler (€/m2 construido) por Distrito y por Trimestre”.
(TEX)

S18 Table Placebo test. Starbucks’ openings at less than 500m. Note: Starbucks’ openings on high-schools’ educational achievement. Authors gathered the information about Starbucks openings and location from the Madrid City Council’s census. Accessible at datos.madrid.es. The authors estimated its distance to high schools.
(TEX)

S19 Table Effect of distance to the closest betting house on academic achievement, in two different levels. Note: Authors’ own elaboration. Data employed originally comes from the Madrid City Council’s census and the education authorities of the Region of Madrid.
(TEX)

S1 Fig Underage commuting patterns in Madrid (2018). Note: Authors own elaboration. Data source: The 2018 Household Mobility Survey conducted by the Consorcio de Mobilidad de Madrid.
(TIF)

S2 Fig Geographic distribution of high schools and betting houses in Madrid (2017). Note: This map was originally created by the authors using open geolocated data from the Madrid City Council and the education authorities of the Madrid Autonomous Community. Stamen Design, under CC BY 4.0, and OpenStreetMap are the sources of the map tiles employed.
(TIF)

S3 Fig Distribution of the high schools’ distance to the closest betting house. Note: Data obtained from the Madrid City Council’s census and the education authorities of the Region of Madrid. The authors’ estimated high schools-betting houses yearly distances.
(TIF)

S4 Fig Evolution of treated and control groups, when using the binary distinction—schools at less than 500m. Plot elaborated using Kim, Rauh, Wang and Imai’s Panelmatch code. Note: Data employed originally comes from the Madrid City Council’s census and the education authorities of the Region of Madrid. The authors’ estimated high schools-betting houses yearly distances.
(TIF)

S5 Fig Results’ summary. Note: Authors’ own elaboration. Data employed originally comes from the Madrid City Council’s census and the education authorities of the Region of Madrid. The authors’ estimated high schools-betting houses yearly logged distances.
(TIF)

S6 Fig Distance to the closest betting house and high-school’s educational performance. Distance is computed in logs. Note: Authors’ own elaboration. Data employed originally comes from the Madrid City Council’s census and the education authorities of the Region of Madrid.
(TIF)

S7 Fig Distance to the closest betting house and high-school’s educational performance. Analyses split by type of school and neighborhood’s average income level. Distance is computed in logs. Note: Analyses split by type of school and neighborhood’s average income level. Distance is computed in logs. Authors’ own elaboration. Data employed originally comes from the Madrid City Council’s census and the education authorities of the Region of Madrid.
(TIF)

S8 Fig Summary of the effect of increasing (reducing) distance to betting houses on educational achievement. Note: Authors’ own elaboration. Data employed originally comes from the Madrid City Council’s census and the education authorities of the Region of Madrid. The authors’ estimated high schools-betting houses yearly logged distances.
(TIF)

S9 Fig Summary of the compositional effect of betting houses. Note: Authors’ own elaboration. Data employed originally comes from the Madrid City Council’s census and the education authorities of the Region of Madrid.
(TIF)

S10 Fig Short-term effect of betting houses on rental prices. (TIF)

S11 Fig Placebo test: Distance to Starbucks. Association between distance to Starbucks coffee shops and educational achievement. Distance is computed in log meters. Note: Authors gathered the information about Starbucks openings and location from the Madrid City Council’s census. Accessible at datos.madrid.es. The authors estimated its distance to high schools.
(TIF)

S12 Fig Placebo test. Starbucks’ openings at less than 500m. Effect of Starbucks’ openings on educational achievement Note: Authors gathered the information about Starbucks openings and location from the Madrid City Council’s census. Accessible at datos.madrid.es. The authors estimated its distance to high schools.
(TIF)

S13 Fig Main effect. Effect of BH openings on HS average grade using the Callaway-Sant’Anna estimator. Note: Authors’ own elaboration. Data employed originally comes from the Madrid City Council’s census and the education authorities of the Region of Madrid.
(TIF)

S14 Fig Compositional change. Effect of BH openings on the number of students using the Callaway-Sant’Anna estimator. Note: Authors’ own elaboration. Data employed originally comes from the Madrid City Council’s census and the education authorities of the Region of Madrid.
(TIF)

S15 Fig Differential effect of betting house on public compared to charter high schools. Note: Authors’ own elaboration. Data employed originally comes from the Madrid City Council’s census and the education authorities of the Region of Madrid.
(TIF)

S1 Text (TEX)

S1 File (PDF)
