# Supplementary figures and images for: The negative consequences of sports betting opportunities on human capital formation: Evidence from Spain
Source: PLoS One. 2021 Oct 27;16(10):e0258857. doi: 10.1371/journal.pone.0258857 (PMC8550418; doi:10.1371/journal.pone.0258857)

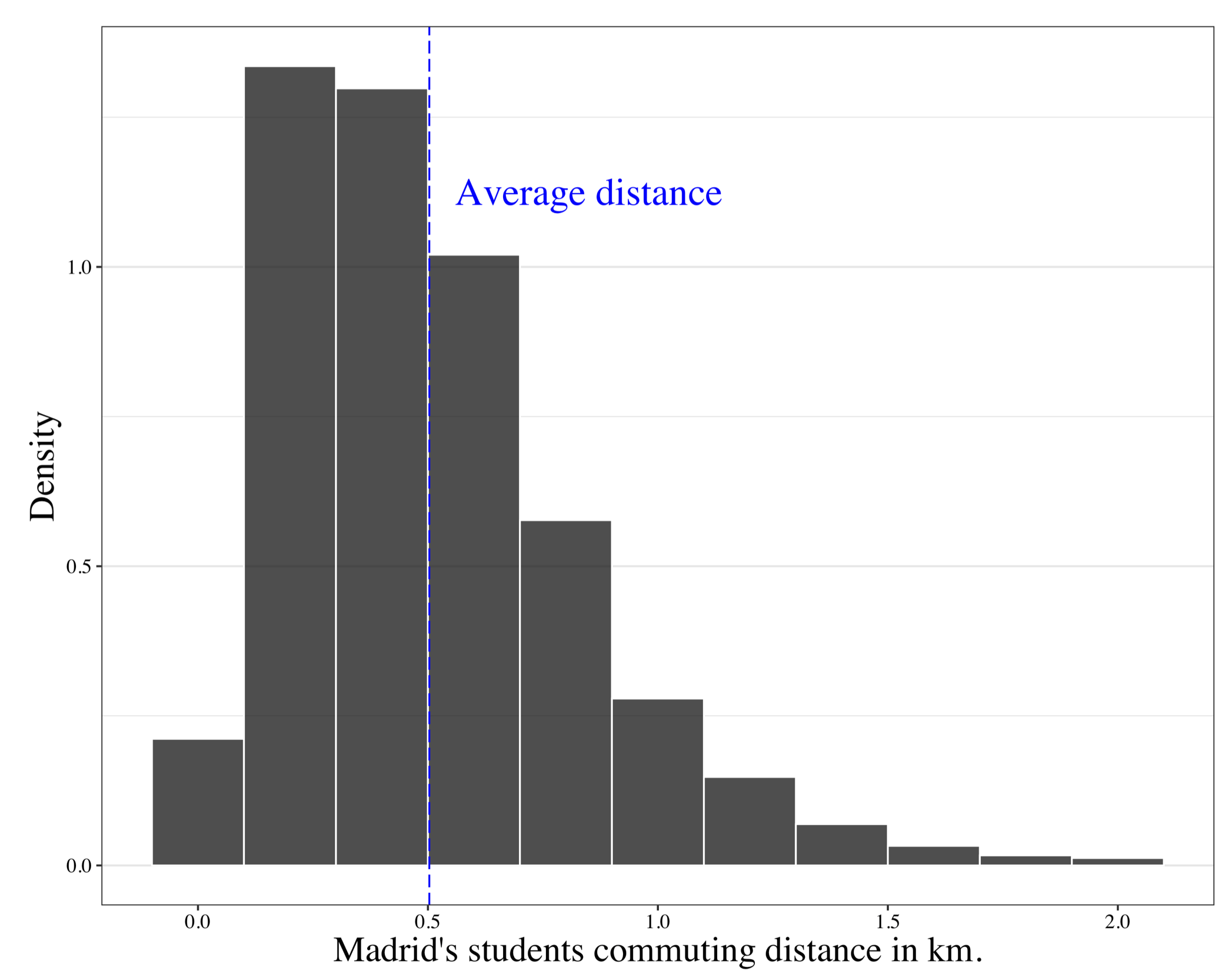

Supplement: S1 Fig — Note: Authors own elaboration. Data source: The 2018 Household Mobility Survey conducted by the Consorcio de Mobilidad de Madrid. (TIF) [file pone.0258857.s020.tif]

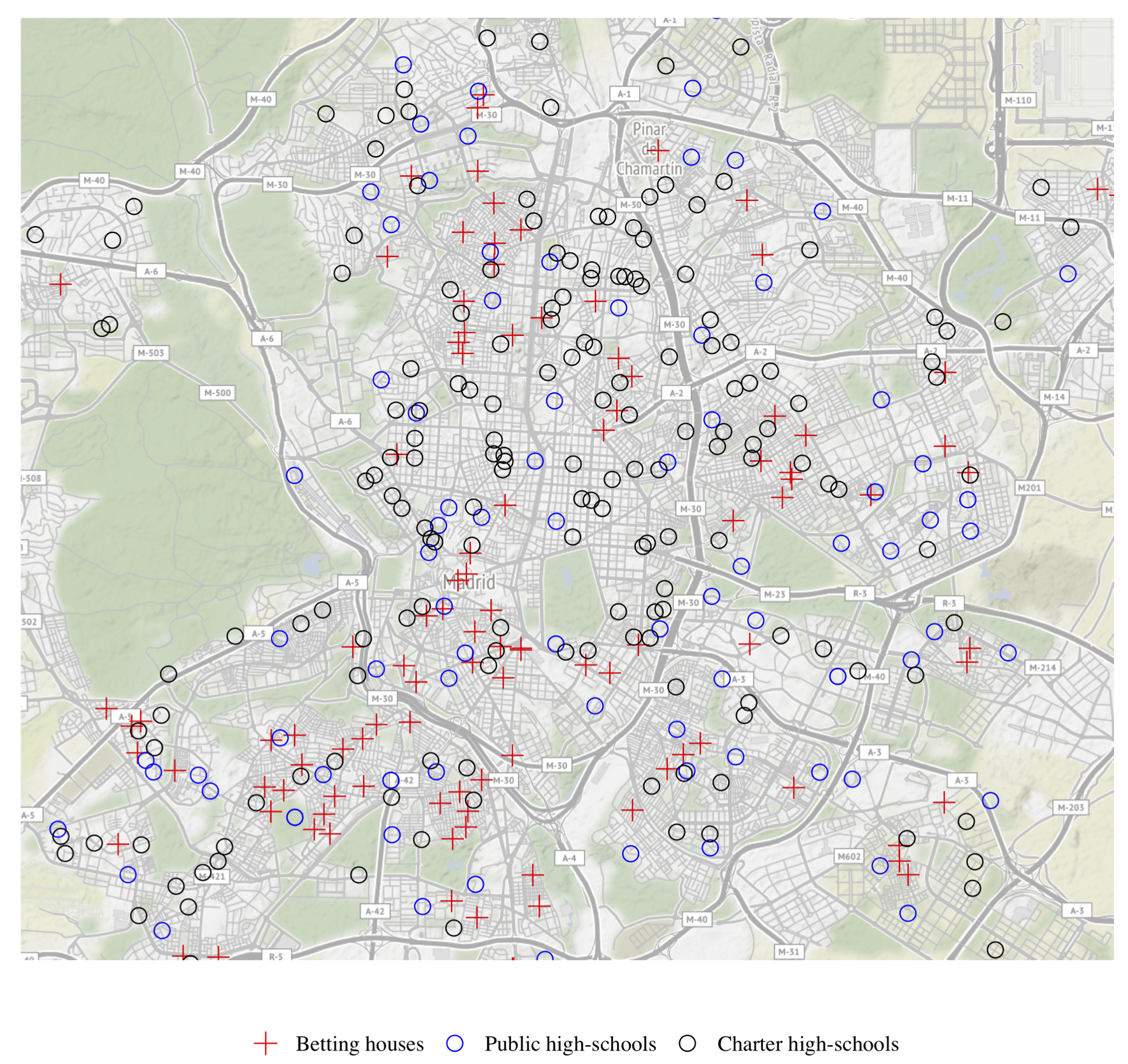

Supplement: S2 Fig — Note: This map was originally created by the authors using open geolocated data from the Madrid City Council and the education authorities of the Madrid Autonomous Community. Stamen Design, under CC BY 4.0, and OpenStreetMap are the sources of the map tiles employed. (TIF) [file pone.0258857.s021.tif]

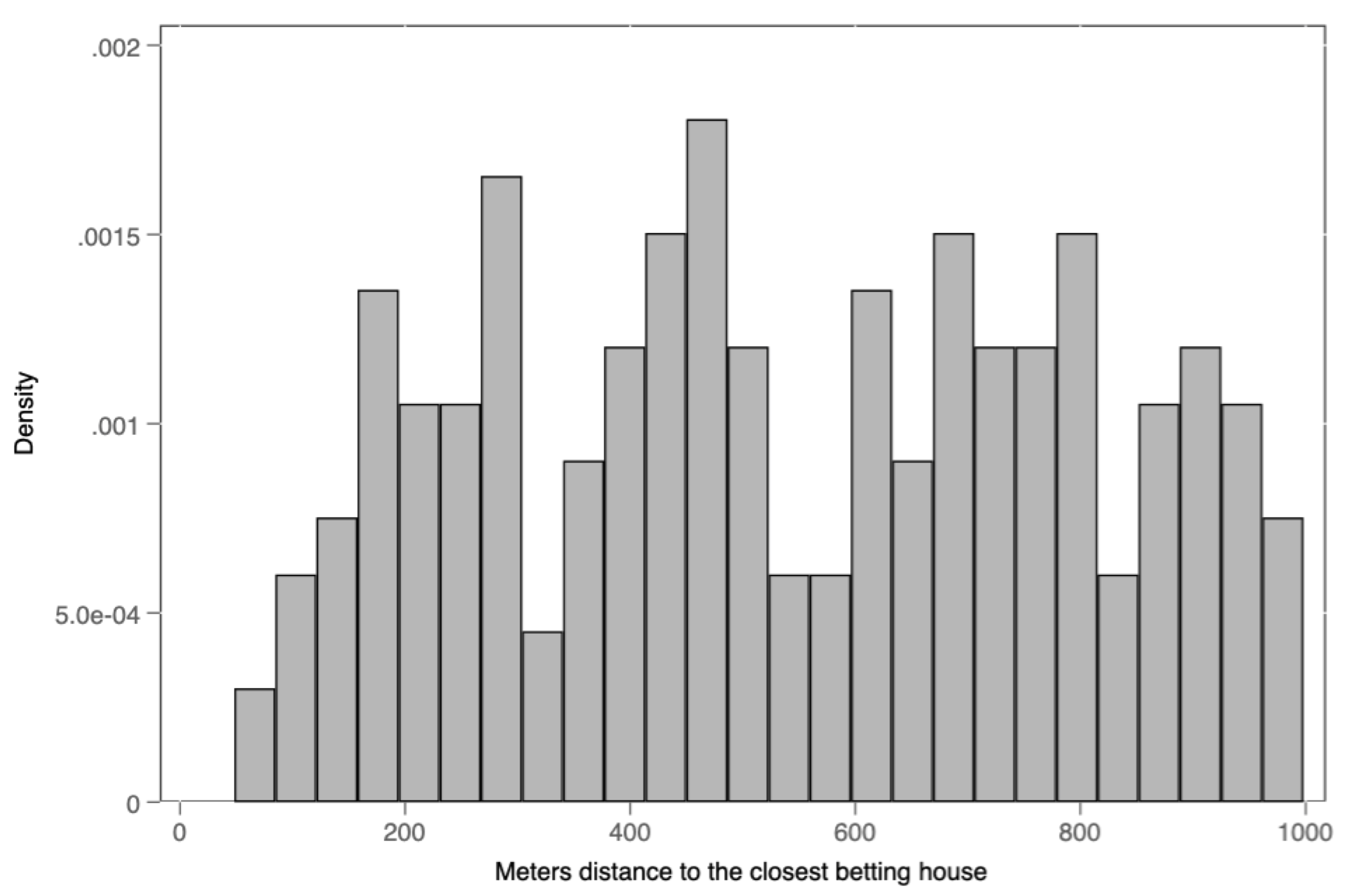

Supplement: S3 Fig — Note: Data obtained from the Madrid City Council’s census and the education authorities of the Region of Madrid. The authors’ estimated high schools-betting houses yearly distances. (TIF) [file pone.0258857.s022.tif]

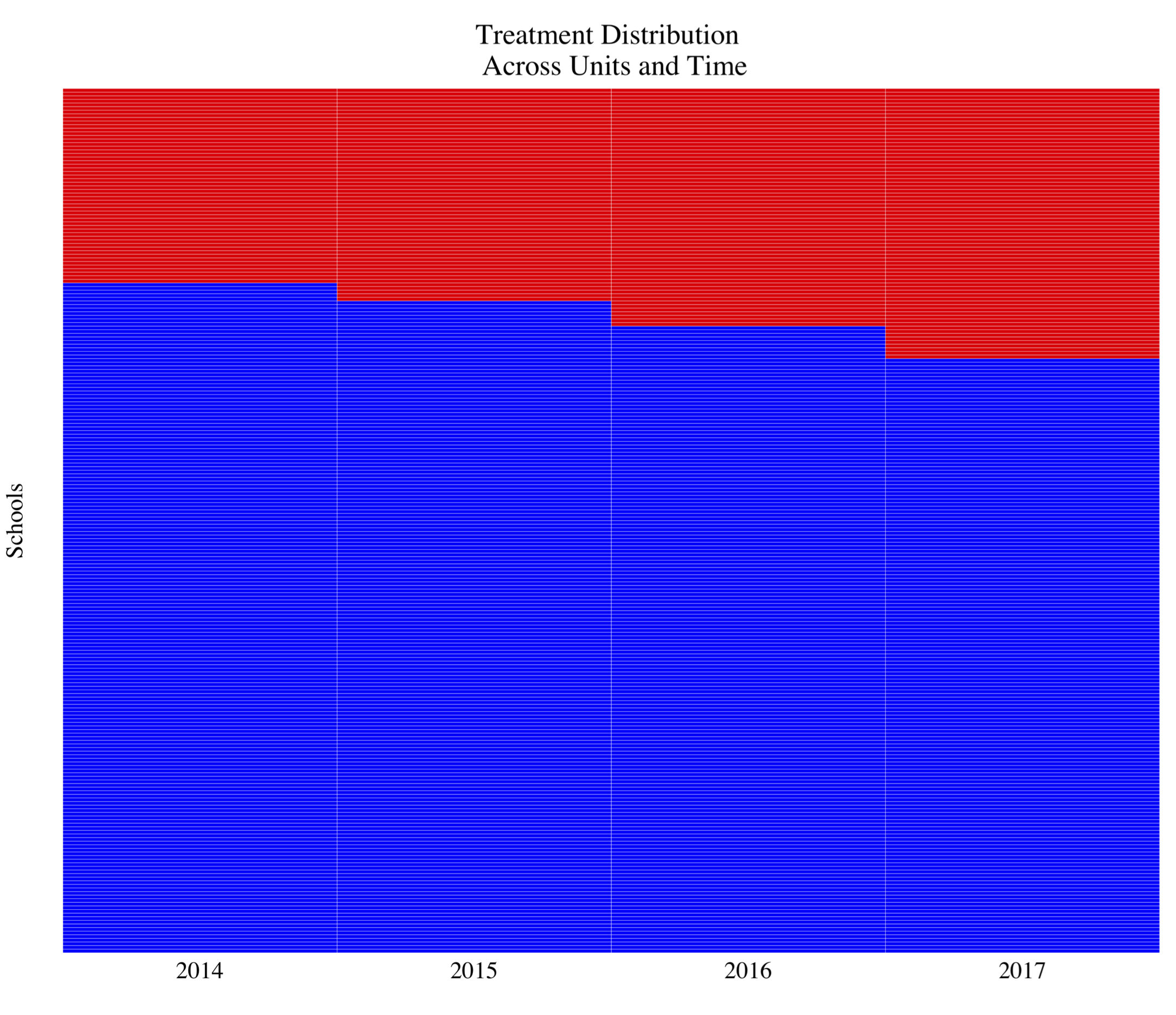

Supplement: S4 Fig — Plot elaborated using Kim, Rauh, Wang and Imai’s Panelmatch code. Note: Data employed originally comes from the Madrid City Council’s census and the education authorities of the Region of Madrid. The authors’ estimated high schools-betting houses yearly distances. (TIF) [file pone.0258857.s023.tif]

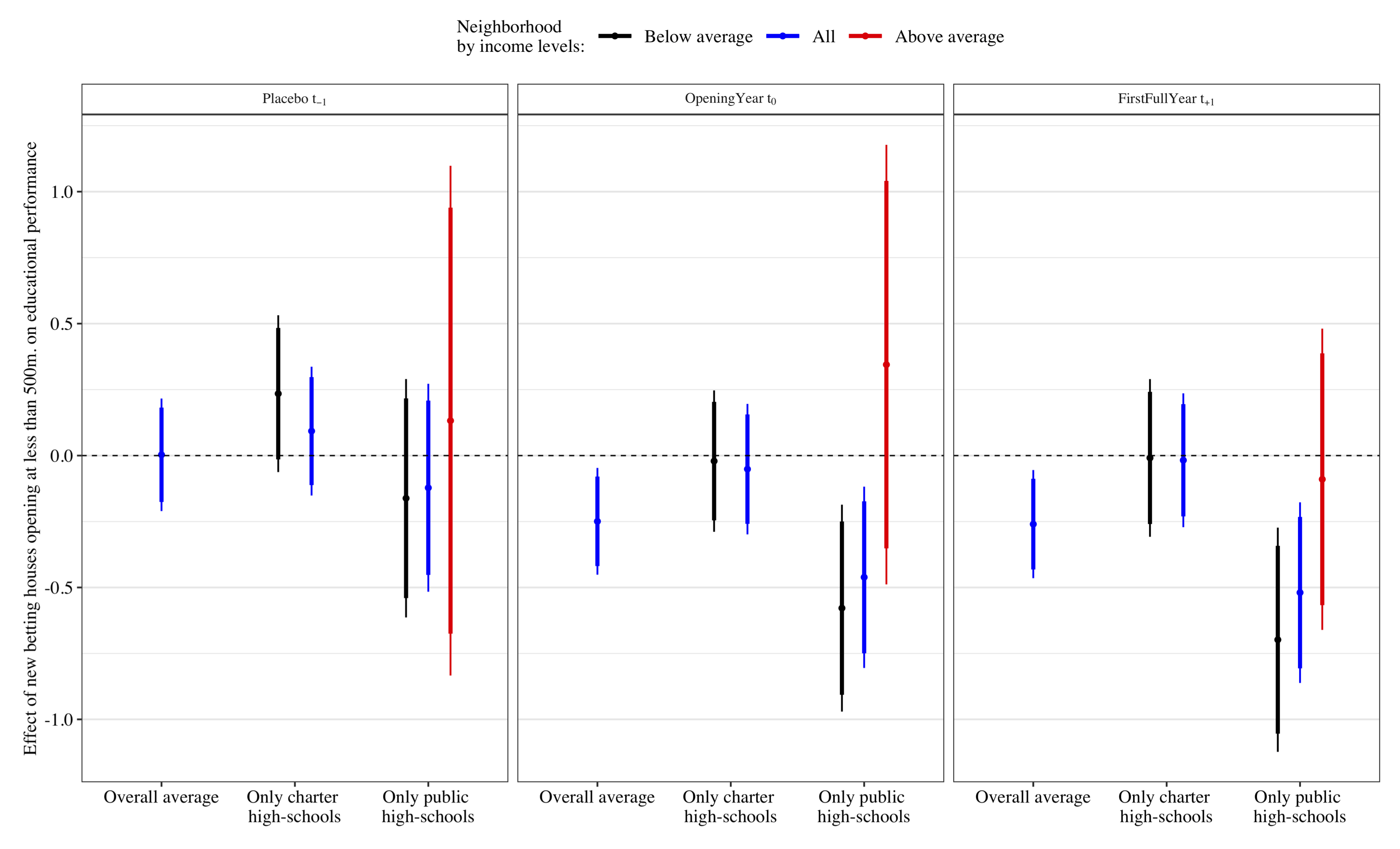

Supplement: S5 Fig — Note: Authors’ own elaboration. Data employed originally comes from the Madrid City Council’s census and the education authorities of the Region of Madrid. The authors’ estimated high schools-betting houses yearly logged distances. (TIF) [file pone.0258857.s024.tif]

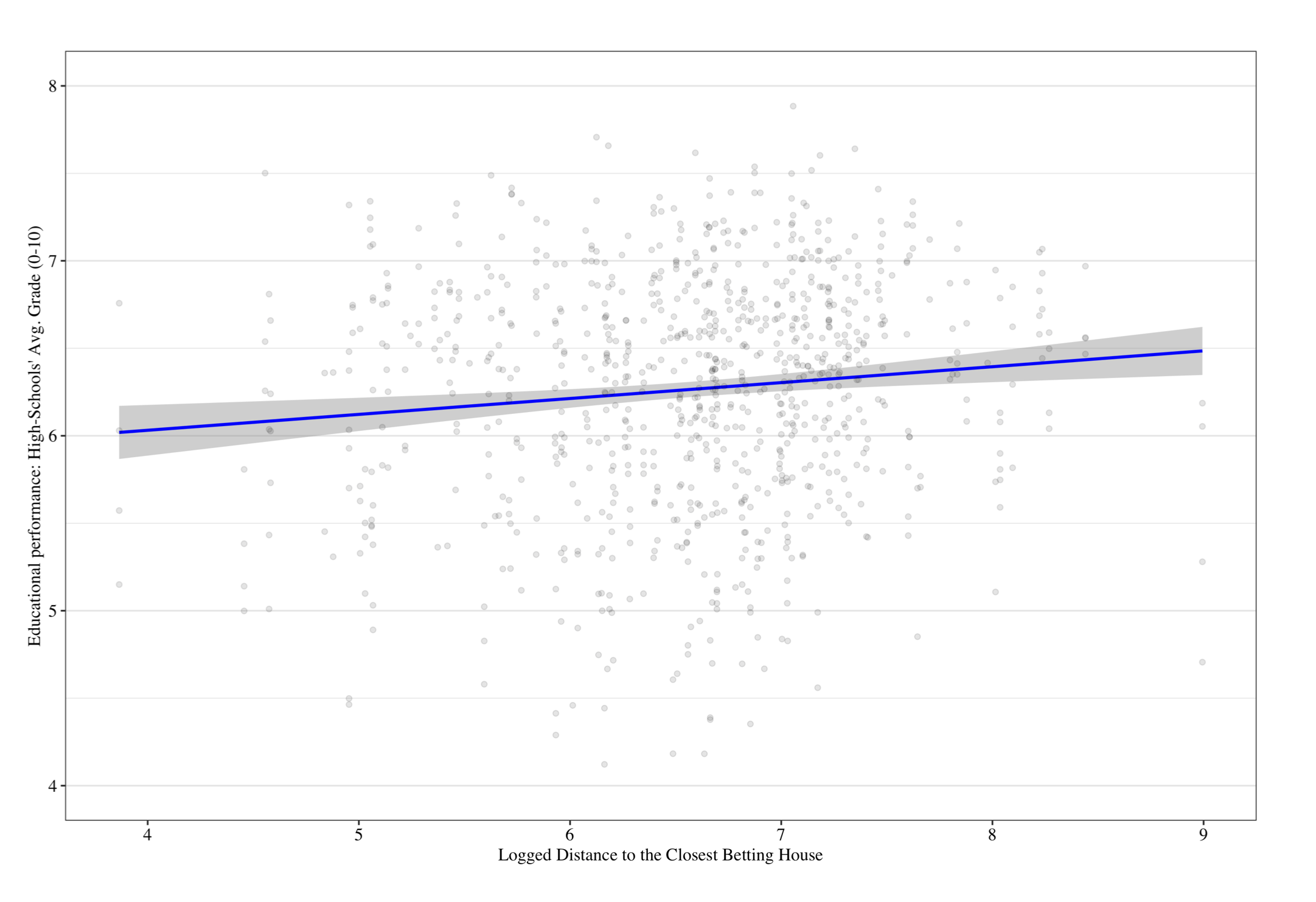

Supplement: S6 Fig — Distance is computed in logs. Note: Authors’ own elaboration. Data employed originally comes from the Madrid City Council’s census and the education authorities of the Region of Madrid. (TIF) [file pone.0258857.s025.tif]

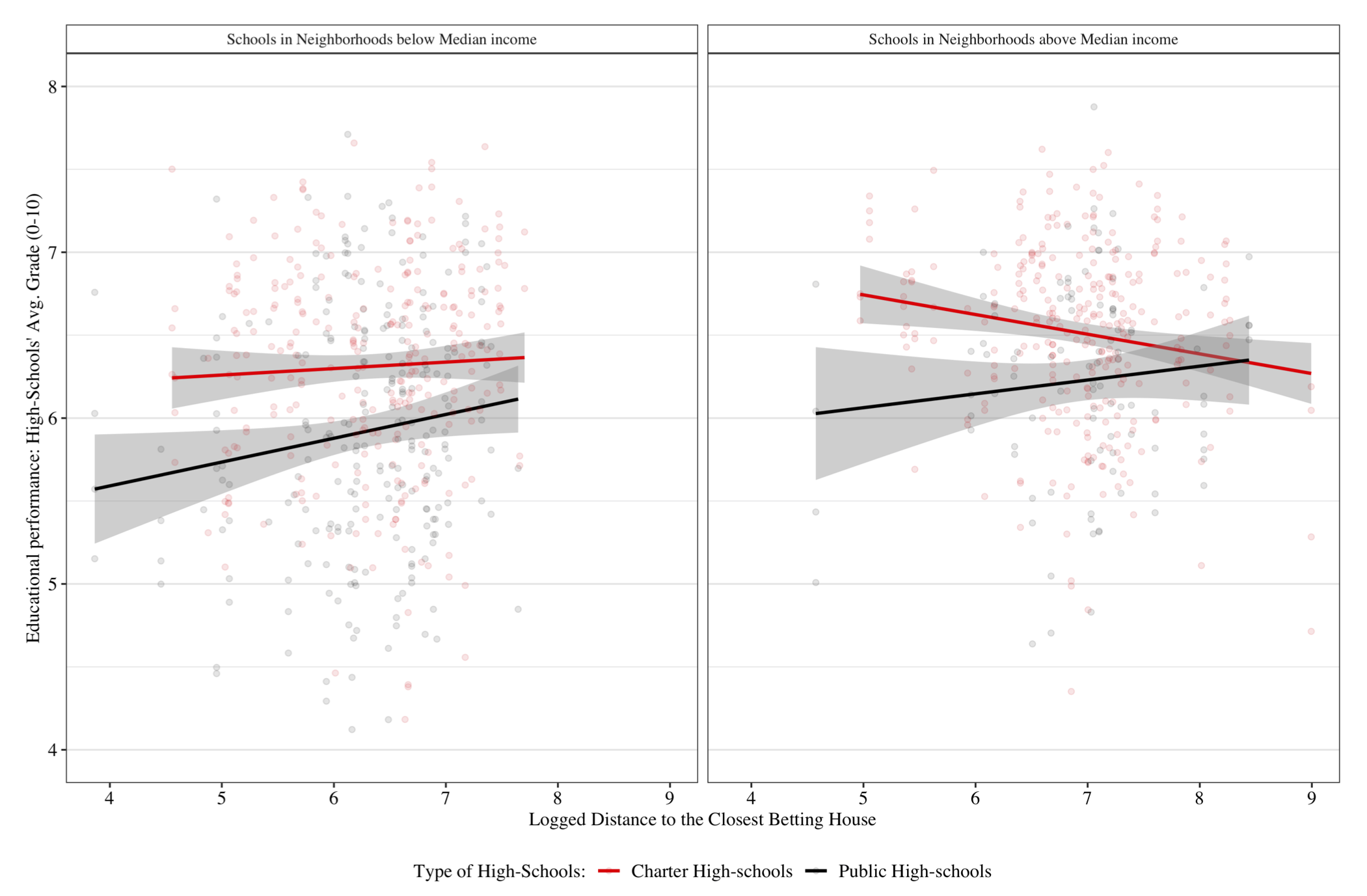

Supplement: S7 Fig — Analyses split by type of school and neighborhood’s average income level. Distance is computed in logs. Note: Analyses split by type of school and neighborhood’s average income level. Distance is computed in logs. Authors’ own elaboration. Data employed originally comes from the Madrid City Council’s census and the education authorities of the Region of Madrid. (TIF) [file pone.0258857.s026.tif]

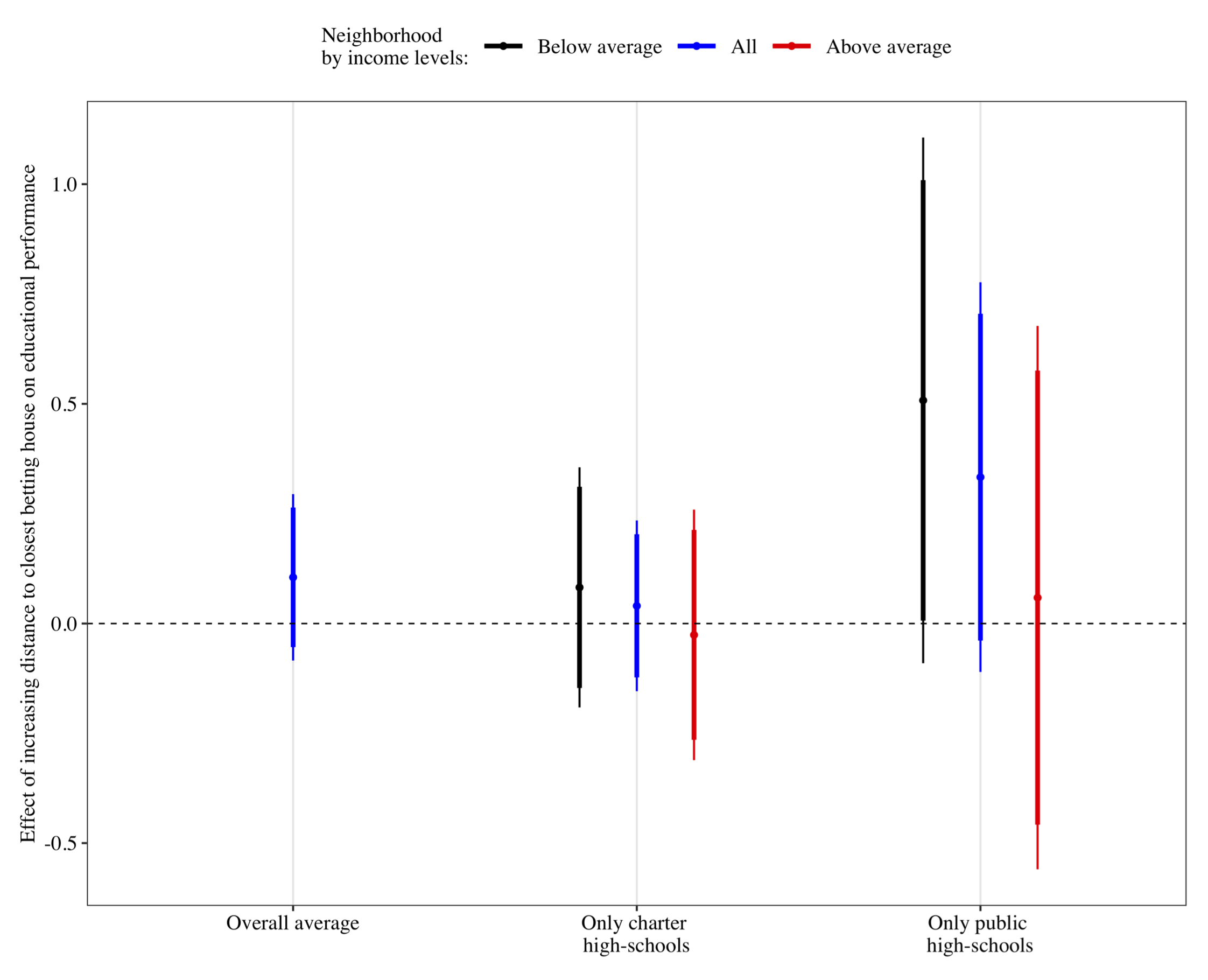

Supplement: S8 Fig — Note: Authors’ own elaboration. Data employed originally comes from the Madrid City Council’s census and the education authorities of the Region of Madrid. The authors’ estimated high schools-betting houses yearly logged distances. (TIF) [file pone.0258857.s027.tif]

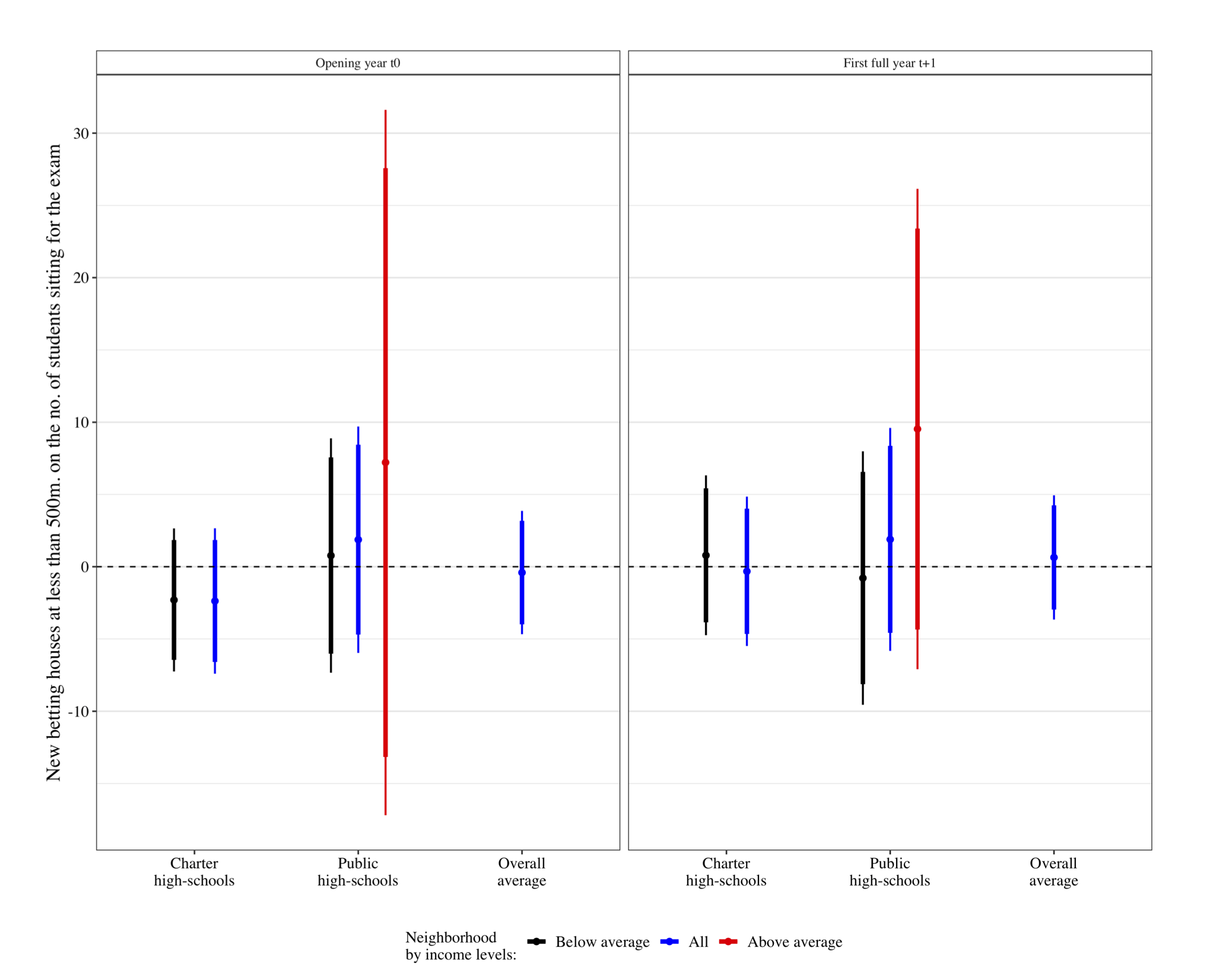

Supplement: S9 Fig — Note: Authors’ own elaboration. Data employed originally comes from the Madrid City Council’s census and the education authorities of the Region of Madrid. (TIF) [file pone.0258857.s028.tif]

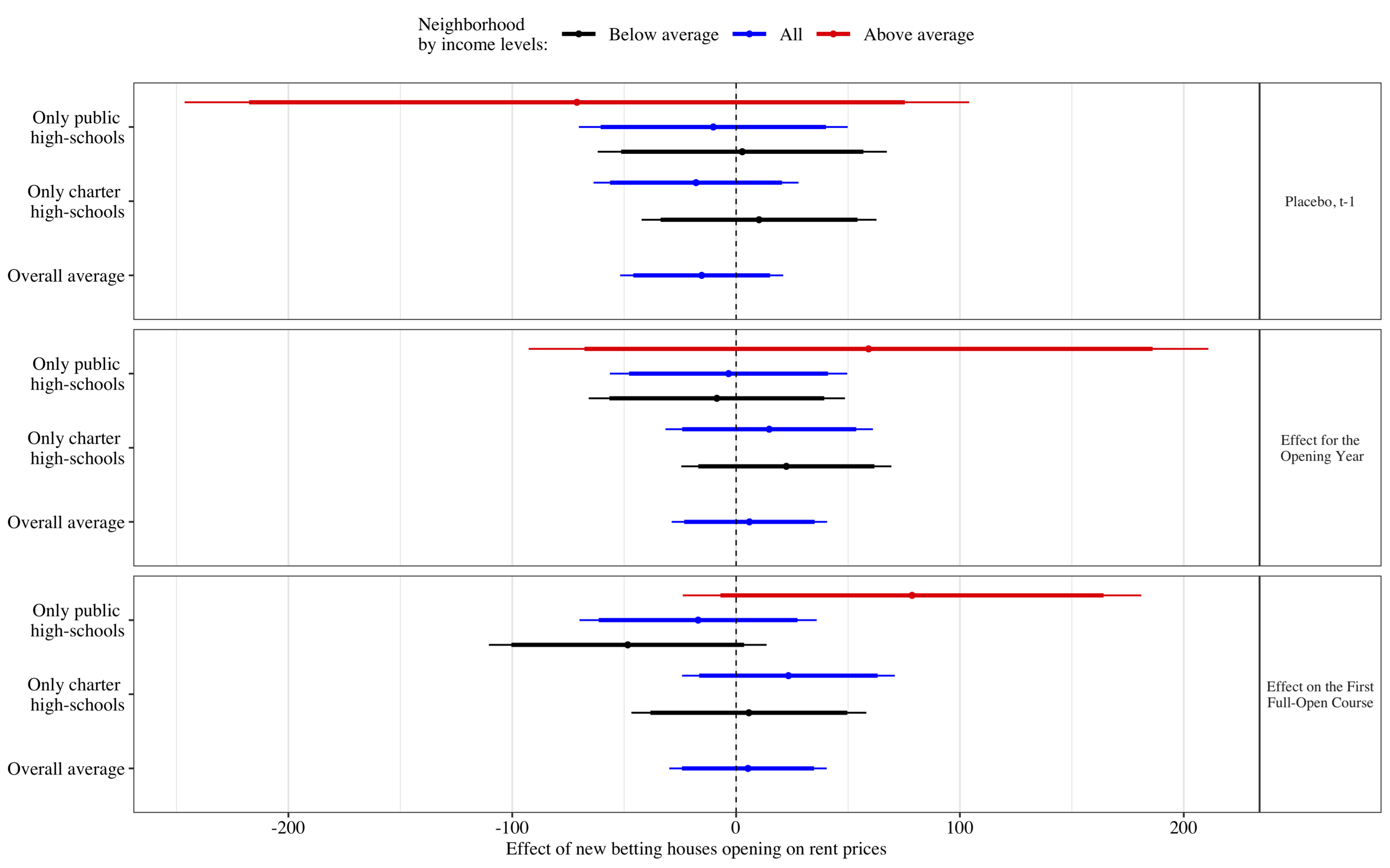

Supplement: S10 Fig — (TIF) [file pone.0258857.s029.tif]

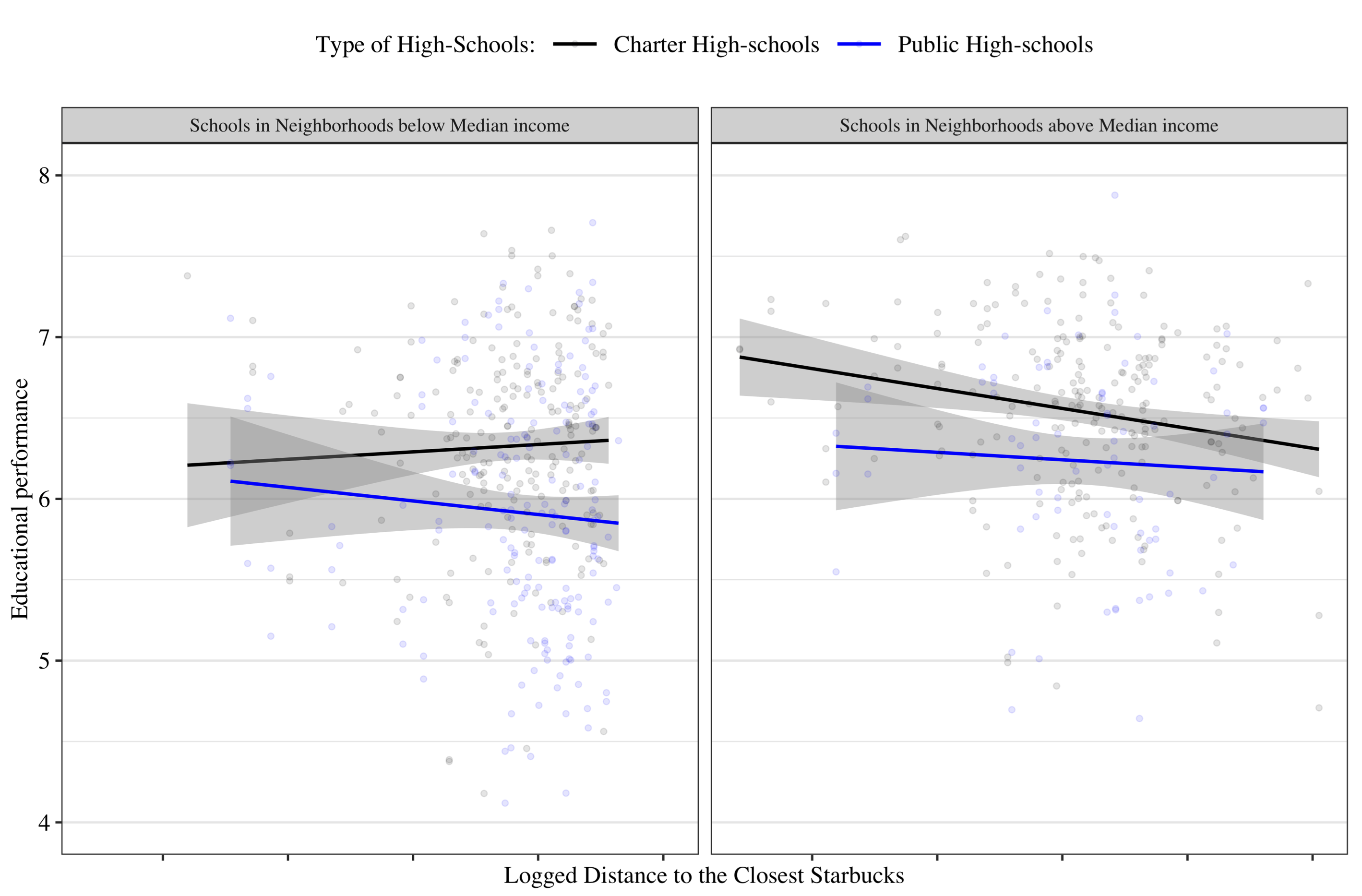

Supplement: S11 Fig — Association between distance to Starbucks coffee shops and educational achievement. Distance is computed in log meters. Note: Authors gathered the information about Starbucks openings and location from the Madrid City Council’s census. Accessible at datos.madrid.es. The authors estimated its distance to high schools. (TIF) [file pone.0258857.s030.tif]

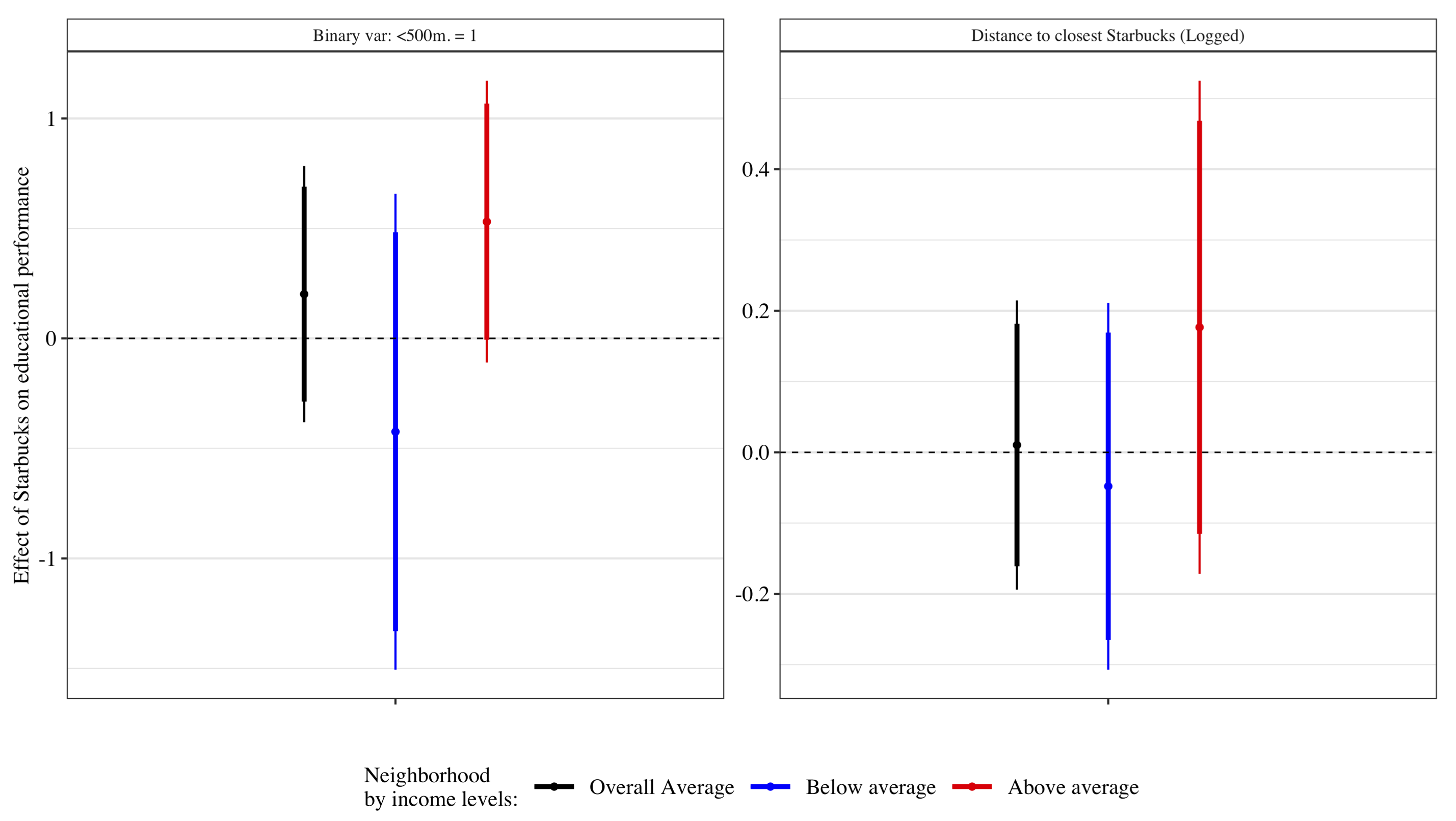

Supplement: S12 Fig — Effect of Starbucks’ openings on educational achievement Note: Authors gathered the information about Starbucks openings and location from the Madrid City Council’s census. Accessible at datos.madrid.es. The authors estimated its distance to high schools. (TIF) [file pone.0258857.s031.tif]

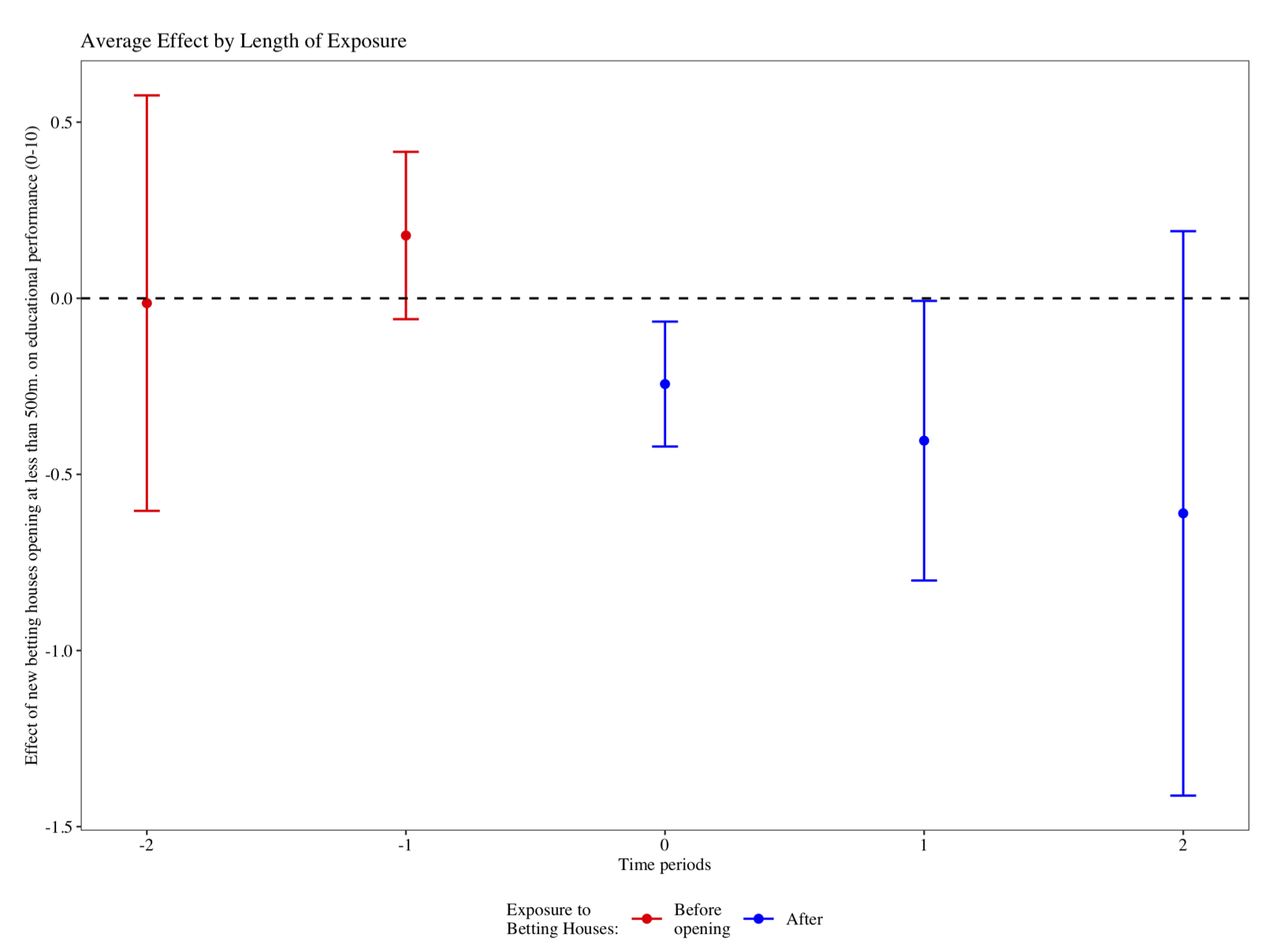

Supplement: S13 Fig — Effect of BH openings on HS average grade using the Callaway-Sant’Anna estimator. Note: Authors’ own elaboration. Data employed originally comes from the Madrid City Council’s census and the education authorities of the Region of Madrid. (TIF) [file pone.0258857.s032.tif]

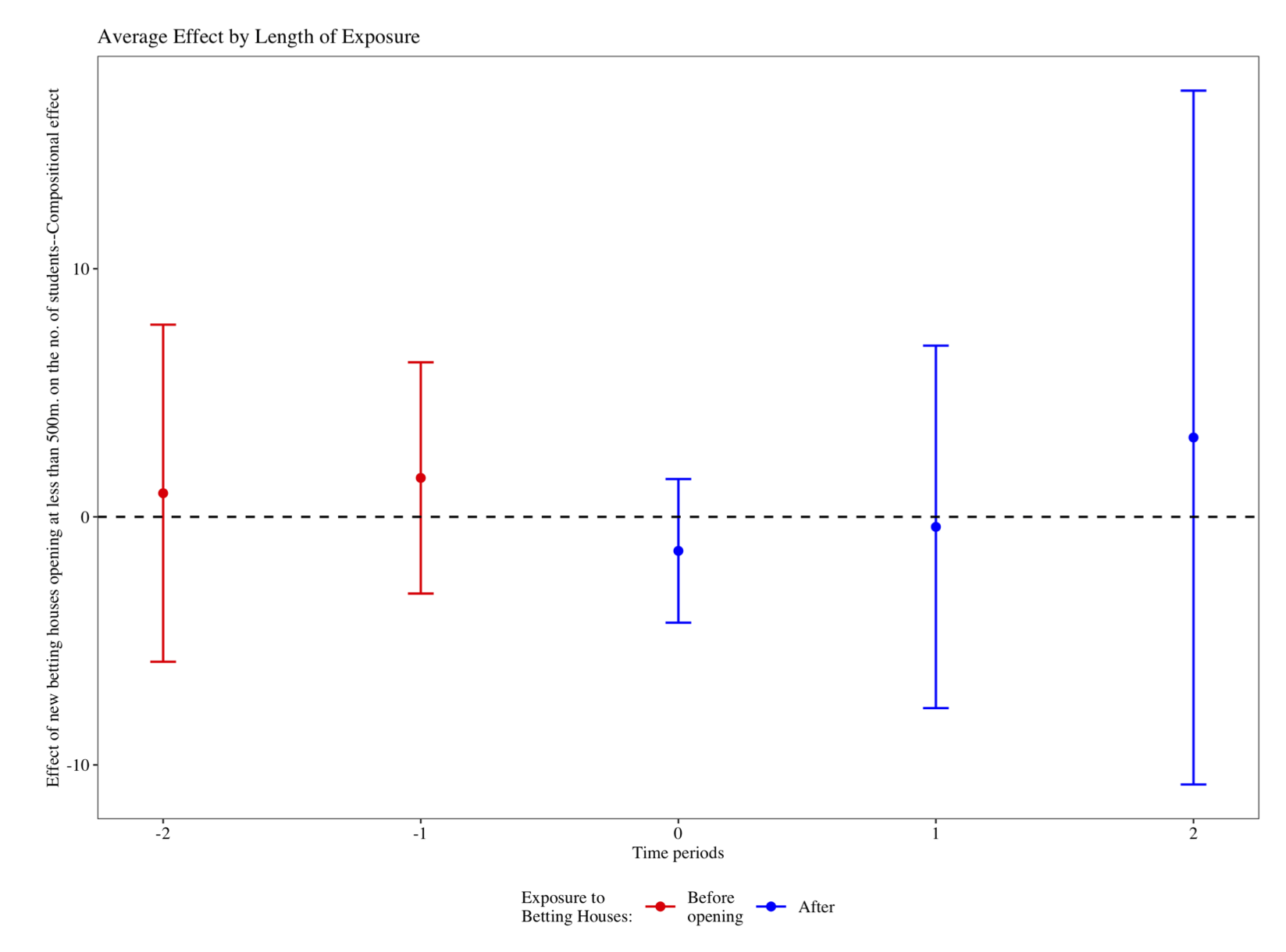

Supplement: S14 Fig — Effect of BH openings on the number of students using the Callaway-Sant’Anna estimator. Note: Authors’ own elaboration. Data employed originally comes from the Madrid City Council’s census and the education authorities of the Region of Madrid. (TIF) [file pone.0258857.s033.tif]

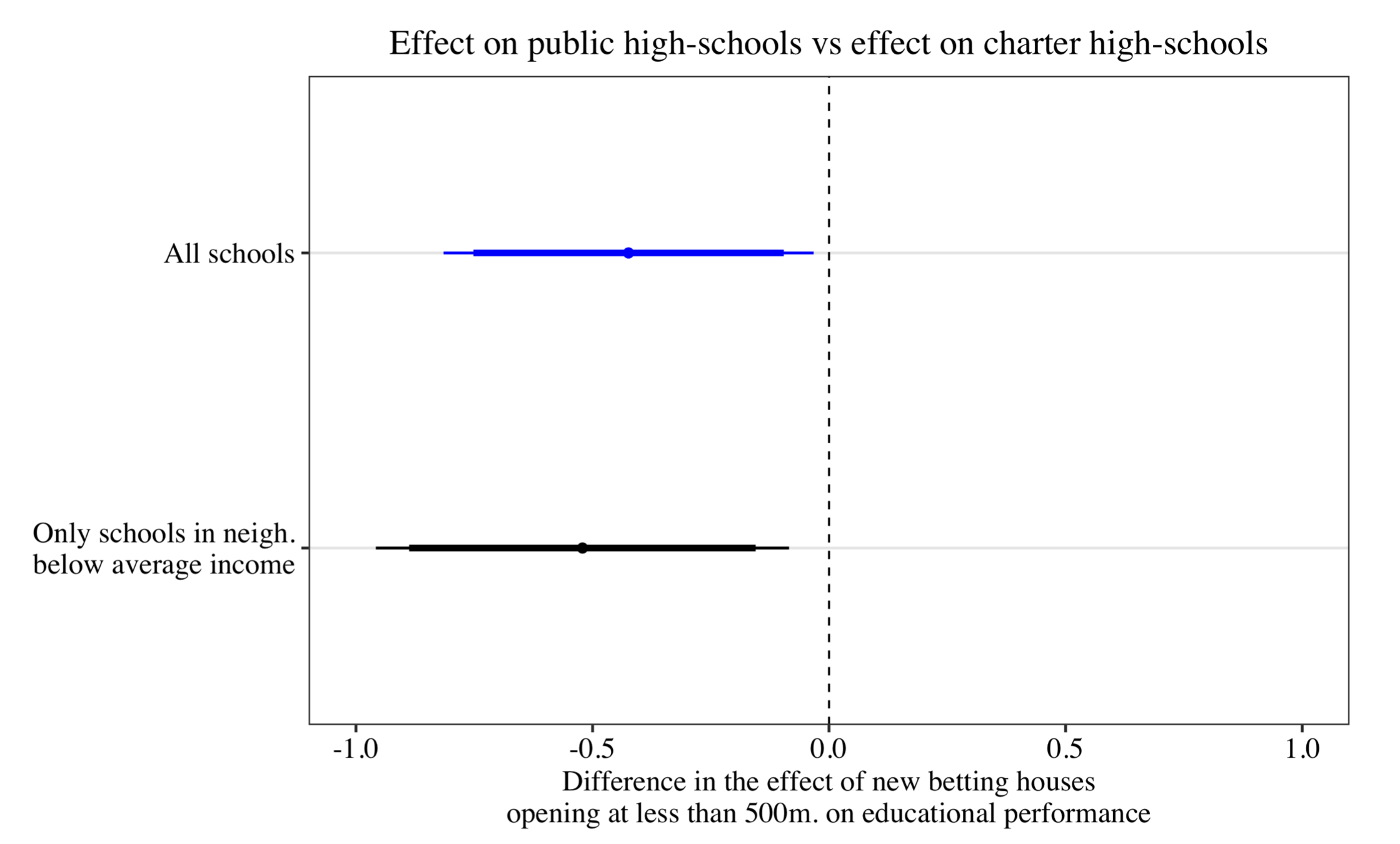

Supplement: S15 Fig — Note: Authors’ own elaboration. Data employed originally comes from the Madrid City Council’s census and the education authorities of the Region of Madrid. (TIF) [file pone.0258857.s034.tif]
