# Supplementary material for: The negative consequences of sports betting opportunities on human capital formation: Evidence from Spain
Source: PLoS One. 2021 Oct 27;16(10):e0258857. doi: 10.1371/journal.pone.0258857 (PMC8550418; doi:10.1371/journal.pone.0258857)
Supplement: S1 File — (PDF) [file pone.0258857.s036.pdf]

Supporting Information for manuscript
"The negative consequences of sports gambling opportunities on human capital formation: evidence from Spain"

Data description

This section details the source and summary statistics of the data employed in this study.

This study uses several sources for data. Information on schools' characteristics and educational performance come from public administrative data yearly released by Madrid's education authorities, which is available *here*. Information on openings of betting houses is available as part of the census data provided by the Government of Madrid, which is also accessible *here*.

We restrict the sample to educational centers that offered the academic track in post-compulsory education (Bachillerato, in Spanish). We obtained information on whether centers were privately or publicly funded, their location, their average grade obtained by each high school at the State-level exams, which grants access to university, and the number of students attending each year. A version of this data has been published and it is publicly available to download here. Additionally, we extracted and linked the average household income level of the neighbourhood where high schools are located, which is also publicly available *here*.

Information about the betting houses was extracted by the authors using the licenses issued by the Government of Madrid to betting houses companies. These licenses come from the following census record: 'Censo de locales, sus actividades y terrazas de hostelería y restauración'. We restricted our sample to private gambling facilities, excluding stores regulated by *Loterías y Apuestas del Estado*, the State-owned enterprise in charge of the most popular both football and lottery games. We coded each license's location and the year in which it was issued to match betting houses to high schools. After this, we calculated walking distances in meters from each high school to the closest betting house to assess each high school's access to gambling facilities. We employ a radius to divide Madrid high schools using a dummy variable switching on those education centers exposed to a betting house at less than 500m. The rationale behind this choice is that, according to the 'Encuesta Domiciliaria de Mobilidad 2018' (Consorcio de Mobilidad de Madrid, Comunidad de Madrid), the average Madrid students' commuting to educational centers is 500 meters, as suggested by descriptive statistics shown in Table. S.1 and Figure. S.1.

S1 Table. Madrid underage commuting summary statistics.
Note: Authors' own elaboration. Data source: The 2018 Household Mobility Survey con-

Variable	Mean	Std. Dev.	Min.	Max.	N
Students' under 18 commuting distance	503.72	323.296	0	1,968.813	5,650

ducted by the Consorcio de Mobilidad de Madrid.

S1 Fig. Underage commuting patterns in Madrid (2018).
Note: Authors' own elaboration. Data source: The 2018 Household Mobility Survey conducted by the Consorcio de Mobilidad de Madrid.

As part of the placebo tests, we extracted licenses issued to Starbucks from the same 'Censo de locales, sus actividades y terrazas de hostelería y restauración'. We then geolocated them and calculated walking distances from each high school to the closest Starbucks. Finally, as part of the robustness checks, we used monthly rent prices by districts. We linked each high school to the rent prices in their area. The data on rent prices can be accessed and downloaded *here*.

Summary statistics

S2 Fig. Geographic distribution of high schools and betting houses in Madrid (2017).
Note: This map was originally created by the authors using open geolocated data from the Madrid City Council and the education authorities of the Madrid Autonomous Community. Stamen Design, under CC BY 4.0, and OpenStreetMap.

S2 Table. Summary statistics.

Variable	Mean	Std. Dev.	Min.	Max.	N
**Outcomes**					
Avg. Grade	6.26	0.70	2.66	8.35	922
Avg. Number of students	46.76	39.17	1	364	862
**Exposure to Betting Houses**					
Binary: Less than 500m.	0.29	0.45	0	1	960
Continuous: Logged meters	6.57	0.80	3.86	8.99	956
**Type of high schools**					
Public H.S.	0.37	0.48	0	1	960
H.S. in areas above median income	0.41	0.49	0	1	960

Note: Authors' own elaboration. Data obtained from the Madrid City Council's census and the education authorities of the Region of Madrid. The authors' estimated high schools-betting houses yearly distances.

S3 Fig. Distribution of the high schools' distance to the closest betting house.
Note: Authors' own elaboration. Data obtained from the Madrid City Council's census and the education authorities of the Region of Madrid. The authors' estimated high schools-betting houses yearly distances.

S3 Table. High schools' distance to the closest betting house.

Year	N	Avg. Distance	St. Dev.	Min	Max
2014	239	1018.85	852.72	47.70	8056.88
2015	239	999.08	856.68	47.70	8056.88
2016	239	936.86	845.47	47.70	8056.88
2017	239	907.57	841.41	47.70	8056.88

Note: Authors' own elaboration. Data obtained from the Madrid City Council's census and the education authorities of the Region of Madrid. The authors' estimated high schools-betting houses yearly distances.

S4 Fig. Evolution of treated and control groups, when using the binary distinction—schools at less than 500m. Plot elaborated using Kim, Rauh, Wang and Imai's Panelmatch code.
Note: Authors' own elaboration. Data employed originally comes from the Madrid City Council's census and the education authorities of the Region of Madrid. The authors' estimated high schools-betting houses yearly distances.

S4 Table. High schools' likelihood of begin exposed to betting houses at less than 500m. Comparison of public and charter schools.

	Likelihood of being treated		
	(1)	(2)	(3)
Public Schools	0.045	0.045	0.007
	(0.058)	(0.058)	(0.059)
Observations	960	960	960
Contr. by income			✓
Year FE		✓	✓

Standard errors in parentheses
* $p < 0.10$, ** $p < 0.05$, *** $p < 0.01$

Note: Authors' own elaboration. Data employed originally comes from the Madrid City Council's census and the education authorities of the Region of Madrid. The authors' estimated high schools-betting houses yearly distances.

Main results

Average effect and time-placebos

S5 Table. Exposure to close-by betting houses on High schools' average performance.

	HS avg. grade in state exams			
	Placebo		Treatment Effect	
	t_{-2}	t_{-1}	t_0	t_{+1}
	(1)	(2)	(3)	(4)
HS at less than	0.069	0.003	-0.249**	-0.260**
500m. to a Betting house	(0.129)	(0.109)	(0.103)	(0.104)
Observations	922	922	922	922
Year FE	✓	✓	✓	✓
High-school FE	✓	✓	✓	✓

Standard errors in parentheses
* $p < 0.10$, ** $p < 0.05$, *** $p < 0.01$

Note: This table includes the pre-openings placebos and the actual average treatment effect. Authors' own elaboration. Data employed originally comes from the Madrid City Council's census and the education authorities of the Region of Madrid. The authors' estimated high schools-betting houses yearly distances.

S6 Table. Effect of betting houses when setting the treatment one year after opening, in t_{+1}.

	Educational achievement one year after opening, t_{+1}						
	ATE	Public only	Public in low income	Public in high income	Charter only	Charter in low income	Charter in high income
	(1)	(2)	(3)	(4)	(5)	(6)	(7)
HS at less than	-0.260**	-0.519***	-0.698***	-0.089	-0.017	-0.008	0.053
500m. to a Betting house	(0.104)	(0.174)	(0.215)	(0.286)	(0.129)	(0.152)	(0.298)
Observations	922	342	242	100	580	291	289
Year FE	✓	✓	✓	✓	✓	✓	✓
High-school FE	✓	✓	✓	✓	✓	✓	✓

Standard errors in parentheses
* $p < 0.10$, ** $p < 0.05$, *** $p < 0.01$

Note: This table distinguishes schools by type and income level. Authors' own elaboration. Data employed originally comes from the Madrid City Council's census and the education authorities of the Region of Madrid. The authors' estimated high schools-betting houses yearly distances.

S7 Table. Effect of betting houses when setting the treatment in the BH opening year, in t_0.

	Educational achievement on the opening year, t_0						
	ATE	Public only	Public in low income	Public in high income	Charter only	Charter in low income	Charter in high income
	(1)	(2)	(3)	(4)	(5)	(6)	(7)
HS at less than	-0.249**	-0.461***	-0.578***	0.345	-0.051	-0.020	0
500m. to a Betting house	(0.103)	(0.174)	(0.199)	(0.418)	(0.126)	(0.136)	(.)
Observations	922	342	242	100	580	291	289
Year FE	✓	✓	✓	✓	✓	✓	✓
High-school FE	✓	✓	✓	✓	✓	✓	✓

Standard errors in parentheses
* $p < 0.10$, ** $p < 0.05$, *** $p < 0.01$

Note: This table distinguishes schools by type and income level. Authors' own elaboration. Data employed originally comes from the Madrid City Council's census and the education authorities of the Region of Madrid. The authors' estimated high schools-betting houses yearly distances.

S8 Table. Placebo test. Effect of betting houses before BH's opening year, in t_{-1}.

	Educational achievement before the opening year, t_{-1}						
	ATE	Public only	Public in low income	Public in high income	Charter only	Charter in low income	Charter in high income
	(1)	(2)	(3)	(4)	(5)	(6)	(7)
HS at less than	0.0030	-0.122	-0.162	0.132	0.092	0.235	-0.266
500m. to a Betting house	(0.109)	(0.200)	(0.229)	(0.484)	(0.124)	(0.151)	(0.244)
Observations	922	342	242	100	580	291	289
Year FE	✓	✓	✓	✓	✓	✓	✓
High-school FE	✓	✓	✓	✓	✓	✓	✓

Standard errors in parentheses
* $p < 0.10$, ** $p < 0.05$, *** $p < 0.01$

Note: This table distinguishes schools by type and income level. Authors' own elaboration. Data employed originally comes from the Madrid City Council's census and the education authorities of the Region of Madrid.The authors' estimated high schools-betting houses yearly distances.

S9 Table. Placebo test. Effect of betting houses two years before BH's opening year, in t_{-2}.

	Educational achievement two years before the opening year, t_{-2}						
	ATE	Public only	Public in low income	Public in high income	Charter only	Charter in low income	Charter in high income
	(1)	(2)	(3)	(4)	(5)	(6)	(7)
HS at less than 500m. to a Betting house	0.069 (0.129)	-0.161 (0.245)	-0.385 (0.288)	0.958** (0.471)	0.206 (0.145)	0.386* (0.223)	0.047 (0.190)
Observations	922	342	242	100	580	291	289
Year FE	✓	✓	✓	✓	✓	✓	✓
High-school FE	✓	✓	✓	✓	✓	✓	✓

Standard errors in parentheses
* $p < 0.10$, ** $p < 0.05$, *** $p < 0.01$

Note: This table distinguishes schools by type and income level. Authors' own elaboration. Data employed originally comes from the Madrid City Council's census and the education authorities of the Region of Madrid. The authors' estimated high schools-betting houses yearly distances.

S5 Fig. Results' summary
Note: Authors' own elaboration. Data employed originally comes from the Madrid City Council's census and the education authorities of the Region of Madrid. The authors' estimated high schools-betting houses yearly logged distances.

Effect of distance to betting houses

S6 Fig. Distance to the closest betting house and high-school's educational performance. Distance is computed in logs.
Note: Authors' own elaboration. Data employed originally comes from the Madrid City Council's census and the education authorities of the Region of Madrid.

S7 Fig. Distance to the closest betting house and high-school's educational performance.
Note: Analyses split by type of school and neighborhood's average income level. Distance is computed in logs. Authors' own elaboration. Data employed originally comes from the Madrid City Council's census and the education authorities of the Region of Madrid.

S10 Table. TWFE models estimated effect of (logged) distance to the closest betting house on educational achievement.

	High-school's educational achievement						
	ATE	Public only	Public in low income	Public in high income	Charter only	Charter in low income	Charter in high income
	(1)	(2)	(3)	(4)	(5)	(6)	(7)
Logged distance to the closest Betting house	0.105 (0.096)	0.333 (0.225)	0.508* (0.303)	0.058 (0.310)	0.040 (0.098)	0.082 (0.139)	-0.026 (0.145)
Observations	918	342	242	100	576	287	289
Year FE	✓	✓	✓	✓	✓	✓	✓
High-school FE	✓	✓	✓	✓	✓	✓	✓

Standard errors in parentheses
* $p < 0.10$, ** $p < 0.05$, *** $p < 0.01$

Note: This table distinguishes schools by type and income level.Authors' own elaboration. Data employed originally comes from the Madrid City Council's census and the education authorities of the Region of Madrid. The authors' estimated high schools-betting houses yearly logged distances.

S8 Fig. Summary of the effect of increasing (reducing) distance to betting houses on educational achievement.
Note: Authors' own elaboration. Data employed originally comes from the Madrid City Council's census and the education authorities of the Region of Madrid. The authors' estimated high schools-betting houses yearly logged distances.

Alternative explanation: compositional change

If the decrease in educational achievement yield by betting houses would come from best students sorting out from affected high-schools, we should see exposed high-schools also reduced the number of students sitting in. We find no evidence suggesting the presence of a compositional change.

Students sorting?

Average effect on the number of students

S11 Table. Compositional change, alternative explanation: Sorting. Betting houses on the number of students sitting in for the exam.

	HS. no. of students sitting in for the exam		
	t_{-1}	t_0	t_{+1}
	(1)	(2)	(3)
HS at less than	0.705	-0.405	0.642
500m. to a Betting house	(2.322)	(2.172)	(2.187)
Observations	862	862	862
Year FE	✓	✓	✓
High-school FE	✓	✓	✓

Standard errors in parentheses
* $p < 0.10$, ** $p < 0.05$, *** $p < 0.01$

Note: Authors' own elaboration. Data employed originally comes from the Madrid City Council's census and the education authorities of the Region of Madrid.

S12 Table. Compositional change, alternative explanation: Betting houses on the no. of students one year after BH opening, in t_{+1}.

	No. of students one year after opening, t_{+1}						
	ATE	Public only	Public in low income	Public in high income	Charter only	Charter in low income	Charter in high income
	(1)	(2)	(3)	(4)	(5)	(6)	(7)
HS at less than	0.642	1.890	-0.786	9.528	-0.320	0.788	-2.907
500m. to a Betting house	(2.187)	(3.914)	(4.435)	(8.297)	(2.628)	(2.804)	(6.655)
Observations	862	282	202	80	580	291	289
Year FE	✓	✓	✓	✓	✓	✓	✓
High-school FE	✓	✓	✓	✓	✓	✓	✓

Standard errors in parentheses
* $p < 0.10$, ** $p < 0.05$, *** $p < 0.01$

Note: This table distinguishes schools by type and income level. Authors' own elaboration. Data employed originally comes from the Madrid City Council's census and the education authorities of the Region of Madrid.

S13 Table. Compositional change, alternative explanation. Betting houses on the no. of students one year after BH opening, in t_0.

	No. of students in BH's opening year, t_0						
	ATE	Public only	Public in low income	Public in high income	Charter only	Charter in low income	Charter in high income
	(1)	(2)	(3)	(4)	(5)	(6)	(7)
HS at less than 500m. to a Betting house	-0.405	1.870	0.771	7.211	-2.373	-2.301	0
	(2.172)	(3.974)	(4.101)	(12.18)	(2.559)	(2.510)	(.)
Observations	862	282	202	80	580	291	289
Year FE	✓	✓	✓	✓	✓	✓	✓
High-school FE	✓	✓	✓	✓	✓	✓	✓

Standard errors in parentheses
* $p < 0.10$, ** $p < 0.05$, *** $p < 0.01$

Note: This table distinguishes schools by type and income level. Authors' own elaboration. Data employed originally comes from the Madrid City Council's census and the education authorities of the Region of Madrid.

S14 Table. Compositional change, alternative explanation. Betting houses on the no. of students one year after BH opening, in t_{-1}.

	No. of students before BH's opening year, t_{-1}						
	ATE	Public only	Public in low income	Public in high income	Charter only	Charter in low income	Charter in high income
	(1)	(2)	(3)	(4)	(5)	(6)	(7)
HS at less than 500m. to a Betting house	0.705	4.990	4.413	8.807	-1.551	-3.035	2.004
	(2.322)	(4.785)	(4.954)	(14.06)	(2.529)	(2.797)	(5.471)
Observations	862	282	202	80	580	291	289
Year FE	✓	✓	✓	✓	✓	✓	✓
High-school FE	✓	✓	✓	✓	✓	✓	✓

Standard errors in parentheses
* $p < 0.10$, ** $p < 0.05$, *** $p < 0.01$

Note: This table distinguishes schools by type and income level. Authors' own elaboration. Data employed originally comes from the Madrid City Council's census and the education authorities of the Region of Madrid.

S9 Fig. Summary of the compositional effect of betting houses.
Note: Authors' own elaboration. Data employed originally comes from the Madrid City Council's census and the education authorities of the Region of Madrid.

Degrading neighborhoods in the short-run?

We remain agnostic about the medium and long-run effect of betting houses on rental prices and the value of neighborhoods. Nevertheless, using rent prices data extracted from open access records—"Renta mensual de la vivienda en alquiler (€/m2 construido) por Distrito y por Trimestre"—, we do not find such an impact in the short-run.

S15 Table. Compositional change. Betting houses on district rent prices in euros/m2, in t_{+1}. This table distinguishes schools by type and income level.

	District rent prices in euros/m2, t_{+1}						
	ATE (1)	Public only (2)	Public in low income (3)	Public in high income (4)	Charter only (5)	Charter in low income (6)	Charter in high income (7)
HS (in districts) at less than 500m. to a Betting house	5.29	-16.97	-48.41	78.59	23.37	5.69	97.03*
	(17.917)	(26.90)	(31.44)	(51.365)	(24.18)	(26.62)	(58.04)
Observations	960	356	256	100	604	304	300
Year FE	✓	✓	✓	✓	✓	✓	✓
High-school FE	✓	✓	✓	✓	✓	✓	✓

Standard errors in parentheses
* $p < 0.10$, ** $p < 0.05$, *** $p < 0.01$

Note: Authors' own elaboration. Data employed originally comes from the Madrid City Council's census and the education authorities of the Region of Madrid. The authors also extracted the data on rent prices from Madrid open access records, "Renta mensual de la vivienda en alquiler (€/m2 construido) por Distrito y por Trimestre".

S16 Table. Compositional change. Betting houses on district rent prices in euros/m2, in t_0.

	District rent prices in euros/m2, t_0						
	ATE (1)	Public only (2)	Public in low income (3)	Public in high income (4)	Charter only (5)	Charter in low income (6)	Charter in high income (7)
HS (in districts) at less than 500m. to a Betting house	5.927	-3.376	-8.618	59.17	14.78	22.43	0
	(17.68)	(26.92)	(29.02)	(76.15)	(23.58)	(23.81)	(.)
Observations	960	356	256	100	604	304	300
Year FE	✓	✓	✓	✓	✓	✓	✓
High-school FE	✓	✓	✓	✓	✓	✓	✓

Standard errors in parentheses
* $p < 0.10$, ** $p < 0.05$, *** $p < 0.01$

Note: This table distinguishes schools by type and income level. Authors' own elaboration.

Data employed originally comes from the Madrid City Council's census and the education authorities of the Region of Madrid. The authors also extracted the data on rent prices from Madrid open access records, "Renta mensual de la vivienda en alquiler (€/m2 construido) por Distrito y por Trimestre".

S17 Table. Compositional change. Betting houses on district rent prices in euros/m2, in t_{-1}.

Table S1: Compositional change. Betting houses on district rent prices in euros/m2, in the opening year, t_{-1}.

	District rent prices in euros/m2, t_{-1}						
	ATE<br>(1)	Public only<br>(2)	Public in low income<br>(3)	Public in high income<br>(4)	Charter only<br>(5)	Charter in low income<br>(6)	Charter in high income<br>(7)
HS (in districts) at less than 500m. to a Betting house	-15.38<br>(18.54)	-10.19<br>(30.49)	2.747<br>(32.73)	-71.11<br>(87.90)	-17.91<br>(23.28)	10.24<br>(26.62)	-63.80<br>(47.83)
Observations	960	356	256	100	604	304	300
Year FE	✓	✓	✓	✓	✓	✓	✓
High-school FE	✓	✓	✓	✓	✓	✓	✓

Standard errors in parentheses
* $p < 0.10$, ** $p < 0.05$, *** $p < 0.01$

Note: This table distinguishes schools by type and income level. Authors' own elaboration. Data employed originally comes from the Madrid City Council's census and the education authorities of the Region of Madrid. The authors also extracted the data on rent prices from Madrid open access records, "Renta mensual de la vivienda en alquiler (€/m2 construido) por Distrito y por Trimestre".

S10 Fig. Short-term effect of betting houses on rental prices.

Placebo test: Starbucks

To confirm it is the wrong nature of the leisure model accompanying betting houses, we replicate our analysis using a different store symbolizing a less invasive and negative leisure model: café places. More specifically, we take advantage of the contemporaneous spread of Starbucks cafés in Madrid during 2010-2015, to evaluate the effect of a non-negative leisure model on high-schools achievement. To do so, we gathered information on Starbucks openings and location from the Madrid City Council's census. Accessible at `datos.madrid.es`.As expected, we find no evidence supporting that Starbucks hampen educational achievement.

S11 Fig. Placebo test: Distance to Starbucks. Association between distance to Starbucks coffee shops and educational achievement. Distance is computed in log meters.
Note: Authors gathered the information about Starbucks openings and location from the Madrid City Council's census. Accessible at `datos.madrid.es`. The authors estimated its distance to high schools.

S18 Table. Placebo test. Starbucks' openings at less than 500m.

Table S2: Placebo test. Starbucks' opening on high-schools' educational achievement.

	Educational achievement					
	ATE	Low income	High income	ATE	Low income	High income
	(1)	(2)	(3)	(4)	(5)	(6)
HS at less than 500m. to a Starbucks	0.201	-0.424	0.531			
	(0.296)	(0.549)	(0.325)			
Logged distance to the closest Starbucks				0.010	-0.048	0.177
				(0.104)	(0.132)	(0.177)
Observations	691	397	294	691	397	294
High-school FE	✓	✓	✓	✓	✓	✓
Year FE	✓	✓	✓	✓	✓	✓

Standard errors in parentheses
* $p < 0.10$, ** $p < 0.05$, *** $p < 0.01$

Note: Starbucks' openings on high-schools' educational achievement. Authors gathered the information about Starbucks openings and location from the Madrid City Council's census. Accessible at `datos.madrid.es`. The authors estimated its distance to high schools.

S12 Fig. Placebo test. Starbucks' openings at less than 500m. Effect of Starbucks' openings on educational achievement.
Note: Authors gathered the information about Starbucks openings and location from the Madrid City Council's census. Accessible at `datos.madrid.es`. The authors estimated its distance to high schools.

Robustness checks

Callaway-Sant'Anna Methodology

We replicated the two main models—both using the average grade and the number of high-school students sitting in, employing the Callaway and Sant'Anna (2020) estimator for staggered difference-in-differences designs.

S13 Fig. Main effect. Effect of BH openings on HS average grade using the Callaway-Sant'Anna estimator.
Note: Authors' own elaboration. Data employed originally comes from the Madrid City Council's census and the education authorities of the Region of Madrid.

S14 Fig. Compositional change. Effect of BH openings on the number of students using the Callaway-Sant'Anna estimator.
Note: Authors' own elaboration. Data employed originally comes from the Madrid City Council's census and the education authorities of the Region of Madrid.

S15 Fig. Differential effect of betting house on public compared to charter high schools.
Note: Authors' own elaboration. Data employed originally comes from the Madrid City Council's census and the education authorities of the Region of Madrid.

Is it really about proximity?

S19 Table. Effect of distance to the closest betting house on academic achievement, in two different levels.

Betting houses on public schools	*Avg. Grade at Exam* to Access University (1)
Less than 500m	-0.507***
	(0.153)
Less than 200m	-0.515
	(0.487)
Constant	6.279***
	(0.0351)
Observations	922
Year FE	✓
School FE	✓

Std. errors in parentheses. * $p < 0.10$, ** $p < 0.05$, *** $p < 0.01$

Note: Authors' own elaboration. Data employed originally comes from the Madrid City Council's census and the education authorities of the Region of Madrid.
